# Experimental analysis of roasted and raw turtle butchery and implications for early human cognition and behaviour

**DOI:** 10.1038/s41598-025-31738-z

**Published:** 2025-12-24

**Authors:** Mariana Nabais, Ruth Blasco, Iratxe Boneta, David Gonçalves, Marina Igreja, Valentina Lubrano, Anna Rufà

**Affiliations:** 1 UNIARQ – Centre for Archaeology, University of Lisbon, Lisbon, Portugal; 2 SWAD - South-West Archaeology Digs, Moura, Portugal; 3 IPHES-CERCA – Catalan Institute of Human Palaeoecology and Social Evolution, Tarragona, Spain; 4 Department of History and Art History, Área de Prehistòria, Rovira i Virgili University, Tarragona, Spain; 5 Autónoma University of Madrid, Madrid, Spain; 6 Arqueozoo S.L, Madrid, Spain; 7 LARC - Archaeosciences Laboratory (LARC), Património Cultural I.P, Lisbon, Portugal; 8 Research Centre for Anthropology and Health, Department of Life Sciences, University of Coimbra, Coimbra, Portugal; 9 Centre for Functional Ecology, Laboratory of Forensic Anthropology, Department of Life Sciences, University of Coimbra, Coimbra, Portugal; 10 CIBIO – Research Centre in Biodiversity and Genetic Resources, University of Porto, Porto, Portugal; 11 ICArEHB – Interdisciplinary Center for Archaeology and the Evolution of Human Behaviour, University of Algarve, Faro, Portugal; 12 PACEA UMR 5199, University of Bordeaux, Bordeaux, France

**Keywords:** Experimental archaeology, Taphonomy, Subsistence strategies, Lithic use-wear, FTIR, Chelonids, Ecology, Ecology, Evolution, Zoology

## Abstract

Chelonid exploitation – including tortoises and freshwater turtles – has been increasingly recognised as a significant element of Palaeolithic subsistence in the Mediterranean and Iberian Peninsula. This study offers an experimental assessment of fire’s role in processing these reptiles, contrasting raw and roasted specimens to evaluate impacts on butchery efficiency, surface modifications, skeletal representation and lithic use-wear. The roasting process markedly reduced disarticulation effort and time, irrespective of the operator’s experience. Cut marks and percussion traces were more frequent in raw-processed individuals, while burnt specimens displayed extensive thermal damage, particularly on carapace plates. However, Fourier-Transform Infrared Spectroscopy (FTIR) revealed limited diagnostic potential for low-intensity thermal exposure. Conversely, lithic tools used in processing exhibited macroscopic edge damage and minor polishes, paralleling wear patterns documented in the butchery of other small fauna. These results align with archaeological evidence from multiple Iberian and Mediterranean sites, suggesting a culturally structured practice of in-shell roasting and anatomical disarticulation. The finds highlight fire’s role in labour optimisation and knowledge transmission, supporting broader discussions on small game exploitation and cognitive planning in early human behaviour.

**Supplementary Information:**

The online version contains supplementary material available at 10.1038/s41598-025-31738-z.

## Introduction

Chelonid remains – primarily tortoises (*Testudo hermanni* and *Testudo graeca*), freshwater turtles (*Mauremys leprosa* and *Emys orbicularis*) and occasionally marine turtles – are increasingly recognised as valuable archaeological indicators of small prey consumption across Palaeolithic sites in the Mediterranean and, in particular, the Iberian Peninsula. Once considered marginal due to their limited meat yield, these reptiles have emerged as consistent elements in faunal assemblages from the Early Pleistocene to the Holocene (e.g.^1–9^). Their presence reflects both their accessibility and their integration into structured subsistence behaviours by early human groups. Across Iberian Palaeolithic sites such as Sima del Elefante, Bolomor Cave, Gruta da Figueira Brava or Gruta da Oliveira, as well as Mediterranean Palaeolithic sites including Qesem, Kebara and Hayonim caves, chelonid remains have been identified in stratified deposits associated with clear anthropogenic activity^10–13^.

Within the Iberian Peninsula, Boneta’s^3^ comprehensive review of more than 10,000 chelonid remains from over 80 archaeological sites demonstrates both the frequency and variability of tortoise and freshwater turtle exploitation. It has shown that tortoises are particularly well represented in Palaeolithic contexts, while freshwater turtles appear more frequently in Holocene layers. However, significant overlap and continuity exist across periods, suggesting long-term exploitation of chelonids in both inland and coastal environments. In several sites, modifications to the shell and the presence of heat alterations have led to explanatory hypotheses focusing on potential secondary uses, such as containers, tools or even as ritualistic elements^3,14,15^. This growing body of data reinforces the idea that chelonid use in Iberia and in the Mediterranean Basin in Prehistory was both widespread and culturally embedded.

In parallel, fire use by early humans has been extensively documented across many of the same archaeological contexts where chelonids appear. At the Middle Pleistocene Qesem Cave, fire was regularly employed for cooking and possibly light management, with repeated burning episodes recorded in association with faunal remains, including chelonids^10,16^. Kebara Cave also provides firm evidence of fire-associated faunal processing, including the roasting of *Testudo graeca*, inferred from spatial associations with hearths and burning patterns concentrated on carapace dorsal plates^12^. In Iberian contexts such as Gruta da Oliveira, stratified combustion features co-occur with burnt chelonian elements, suggesting fire was an integral part of daily routines^11,17^. These combustion traces – charcoal layers, reddened sediment and thermally altered bone – support broader arguments about fire’s centrality not only in food processing, but in the organisation of space, sociality and technological transmission during the Palaeolithic^18,19^.

As such, this study investigates the role of fire in the processing of tortoises and freshwater turtles by experimentally comparing raw and roasted chelonid specimens. It explores how thermal alteration affects butchery efficiency, including time investment and tool use required to disarticulate carcasses. Our research documents bone surface modifications – such as cut marks, percussion traces and thermal damage –, as well as differences in skeletal part representation and fragmentation across treatment conditions. In addition, Fourier-Transform Infrared Spectroscopy (FTIR) is employed to characterise the molecular and crystallinity changes in burnt bone, enabling a more precise identification of heat exposure levels. Lithic use-wear analysis was also undertaken to enhance the identification of processing actions involved in the exploitation of tortoises and freshwater turtles. Through a combination of analytical techniques, this study provides a controlled experimental framework for interpreting fire-mediated processing behaviours in the archaeological record, thereby contributing to broader discussions on Palaeolithic subsistence, fire use and the cognitive and cultural dimensions of small game exploitation.

## Methods

### Chelonid taxa

Two freshwater turtles were provided by a private owner in Lisbon (Portugal) and four tortoises were provided by the Amphibian and Reptile Recovery Centre of Catalonia (CRARC in Barcelona, Spain). Both entities gave appropriate permission to use the dead animals for the experimentation presented in this study. The animals died under natural conditions, at home, or at the rehabilitation centre, following veterinary checks. They were, then, frozen aiming at their preservation for scientific research.

Chelonid selection tried to represent species most commonly associated with early human diets in the Iberian Peninsula (e.g.^1,2,5,6,11,20,21^), but their obtainability was dependent of their abundance and natural deaths. Although the tortoise specimens used in this study belong to the same taxonomic species as those recovered from Iberian Palaeolithic sites — namely *Testudo hermanni* (Hermann’s tortoise, Chelonids 3 to 6) —, the same does not apply for the freshwater turtles. The freshwater turtles found in archaeological sites – i.e. *Mauremys leprosa* and *Emys* *orbicularis* – are currently protected and exist in small populations, making them difficult to obtain. Consequently, the absence of the exact same freshwater turtle taxa represents a limitation to our study – particularly in terms of direct taphonomic comparability between archaeological and present-day specimens –, so the most suitable analogues available for freshwater turtles had to be selected. These include *Graptemys ouachitensis* (Ouachita map turtle, Chelonid 1) and *Mauremys* cf. *reevesii* (Chinese pond turtle, Chelonid 2), both of which are non-native species in the Iberian Peninsula (Tab. 1; S.I. 1). This limitation is especially relevant in the case of *Emys orbicularis* which differs morphologically from the experimental specimens, due to a ligamentous connection between the carapace and plastron rather than a bony bridge, a feature likely to influence disarticulation processes, the type of fractures and other marks produced during butchery.

**Table 1:** Summary of chelonid specimens used in the experimental study detailing species name, body measurements (lengths, widths, weights, in centimetres and grams), processing actions undertaken and duration of each experimental processing duration (in minutes), the lithic implements used, the experience of the experimenter and additional observations noted prior and during the experimental procedure.

	CHELONID 1	CHELONID 2	CHELONID 3	CHELONID 4	CHELONID 5	CHELONID 6
Species	*Graptemys ouachitensis*	*Mauremys* c.f. *reevesii*	*Testudo hermanni*	*Testudo hermanni*	*Testudo hermanni*	*Testudo hermanni*
Species habitat	River (Freshwater turtle)	River (Freshwater turtle)	Land (Tortoise)	Land (Tortoise)	Land (Tortoise)	Land (Tortoise)
Carapace length	14.55 cm	9.31 cm	15.36 cm	13.08 cm	10.98 cm	10.38 cm
Carapace width	11.57 cm	6.76 cm	12.61 cm	11.25 cm	8.7 cm	7.87 cm
Plastron length	14.17 cm	8.71 cm	12.96 cm	11.11 cm	9.95 cm	9.49 cm
Plastron width	9.21 cm	5.81 cm	10.74 cm	8.17 cm	7.73 cm	6.57 cm
Weight	353 g	91 g	601 g	419 g	192 g	171 g
Actions	Raw	Roasted (2 min carapace + 2 min plastron)	Roasted (5 min carapace + 5 min plastron)	Roasted (5 min carapace + 5 min plastron)	Raw	Raw
Manual processing duration	42 min	4 min	6.50 min	7.27 min	49.12 min	9.20 min
Lithic implements used	Quartzite hammerstone 2a Flint flake 6	Quartzite hammerstone 4a Flint flake 5	Quartzite hammerstone TT9 Flint flake TT5	Quartzite hammerstone TT7 Flint flake TT2	Quartzite hammerstone TT6 Flint flakes TT3 and TT4	Quartzite hammerstone TT8 Flint flake TT1
Experimenter	Non-experienced	Experienced	Experienced	Non-experienced	Non-experienced	Experienced
Observations	Female adult featuring one fully formed egg and another two forming	Young animal based on long bone epiphysis observation	Very light pyramiding on the carapace	At 8 min of roasting, the carapace fractured longitudinally on its left side		

Chelonid species identification was confirmed through macroscopic observations, using the osteological reference collections housed at the Catalan Institute of Human Palaeoecology and Social Evolution (IPHES, Spain) and at the Património Cultural I.P.’s Archaeosciences Laboratory (LARC, Portugal), as well as by applying the diagnostic criteria outlined by Rhodin et al.^22^.

The specimens were left to defrost for 24 h before the start of the experimentation to ensure the carcasses closely resembled their original raw state and appearance. All photographs, weights and measurements (i.e. carapace length, carapace width, plastron length, plastron width) were recorded only after the defrosting process was complete.

### Experimental protocol

Prior to processing for experimental purposes, all chelonids were photographed with a Nikon D5300 and an iPhone11, they were measured with a calliper DURC DC-1 Artikel-Nr.65002697 and weighted with Tristar scale model KW-2446 (Tab.1; S.I. 1, 2). The archaeological experiments involving freshwater turtles were carried out in Lisbon (Portugal), while those with tortoises were conducted in Botarell (Spain). All experiments took place in controlled settings designed to replicate early human methods for processing and cooking chelonid species. Chelonids were prepared, cooked and processed according to archaeological evidence and ethnographic data (e.g.^2,3,10,11,13^).

To gain a deeper insight into various potential consumption practices, three individuals were butchered in their raw state (*Graptemys ouachitensis* and *Testudo hermanni*, corresponding to Chelonids 1, 5 and 6), whereas the other three were butchered after cooking (*Mauremys* c.f. *reevesii* and *Testudo hermanni*; or Chelonids 2, 3 and 4; Tab. 1; S.I. 1), by three different people with distinctive experiences in carcass processing. Operators with no prior experience processing chelonids, either raw or roasted, are classified non-experienced, while those with previous butchering experience are considered as experienced.

The three raw chelonid specimens were manually butchered, with no pre-treatment of the individuals. The remaining three specimens were cooked by roasting directly on embers, with the temperature ranging between 448ºC and 640ºC, depending on the specific location of measurement on the coals. This temperature range was selected to produce black-coloured burn marks like those observed archaeologically, which are considered to result from exposure to heat between 300ºC and 645ºC^23,^^24^. Coal was used as the fuel source for the fire, rather than wood as may have been the case in prehistoric contexts. However, it is assumed that the resulting embers would have produced similar black burning marks, making the outcome comparable. The specimens were cooked intact, without any prior breakage or dissection before heat exposure.

The temperature was consistently monitored using an Infrared Thermometer MS model GENMSPL. Whole chelonid specimens were initially roasted with their plastron facing upwards and the carapace in direct contact with the embers. Chelonid 2 was cooked in this position for four minutes, Chelonids 3 and 4 were roasted for five minutes. Following this initial phase, the specimens were flipped, placing the plastron in direct contact with the coals for an additional two minutes in the case of Chelonid 2, for five minutes for Chelonids 3 and 4. Cooking durations were established empirically by evaluating the doneness of the chelonid meat, which was observed to cook rapidly when in direct contact with the embers.

The butchering techniques relied on manual handling and the use of lithic tools. Carapaces were fractured using quartzite hammerstones, while skin and ligaments were severed with the aid of experimental flint flakes (Tab. 1; S.I. 1). Evisceration and long bone disarticulation were predominantly achieved through manual movements involving flexion, twisting, pulling and overextension, sometimes aided by flint flakes (S.I. 2). Following processing, the bones of each chelonid specimen were placed in separate laundry bags and submerged in a Neutrase enzyme solution. This method facilitated the removal of soft tissues within a controlled temperature range of 34–55ºC over a period of 3 to 12 h^25^. After enzymatic treatment, the bones were thoroughly cleaned, brushed and sun-dried to deactivate any residual enzymes.

The chelonid remains underwent detailed macroscopic and microscopic analysis using a Hirox HR 5000E at LARC, in Lisbon. This analysis aimed to document bone surface modifications, including cut marks, percussion evidence (e.g., notches and percussion flakes) and their distribution, location and orientation. Additionally, patterns of breakage such as fragment size, fracture outlines, fracture angles and fracture edges were systematically recorded. Since chelonid skeletal remains from Palaeolithic contexts predominantly comprise carapace and plastron fragments, as well as robust appendicular elements, our observations primarily focused on these anatomical components (such as the scapula, coracoid, humerus, ulna, radius, ilium, femur, tibia and fibula).

All experimental procedures were comprehensively recorded, with video files archived in the CORA repository (https://dataverse.csuc.cat/dataset.xhtml?persistentId=doi%3A10.34810%2Fdata2413) to support future reference and potential replication. The corresponding analyses of the processed specimens are likewise accessible within the same repository (https://dataverse.csuc.cat/dataset.xhtml?persistentId=doi%3A10.34810%2Fdata2412).

It is important to acknowledge that the experimental setting may not fully replicate the precise environmental conditions encountered by early humans. Additionally, the butchering techniques employed in this study may not perfectly mimic the exact gestures used by prehistoric individuals. These limitations should be considered when interpreting the results of this research.

### FTIR analysis

A total of 15 samples were collected from three *Testudo hermanni* specimens: Chelonids 3 (roasted), Chelonid 4 (roasted) and Chelonid 6 (processed raw). For each individual, five samples were taken: one from the exterior and one from the interior of the carapace, one from the exterior and one from the interior of the plastron, and one from the shaft of the left femur. Sampling consisted of scraping the bone to obtain a powdered sample. The most superficial scraping was discarded and only the additional scraping from the underground region was used for the analyses. This approach was adopted to minimise any potential diagenetic effects. Powdered samples were kept in Eppendorf tubes.

Spectra were acquired in the mid-infrared region (400–4000 cm-1) using a Bruker ALPHA II FTIR spectrometer coupled to an attenuated total reflectance (ATR) module with diode laser and single-reflection diamond crystal. A deuterated triglycine sulphate detector was used. The correction for the frequency dependence of the incident radiation in ATR mode was made using the Opus 7.2 software. For each spectrum, 64 acquisitions were added, with a resolution of 4 cm-1 and three-term Blackman-Harris apodisation and a background with 64 acquisitions was obtained. The frequency dependence of the penetration depth of the electric field in ATR was corrected through an algorithm from the Opus 8.1 software (using a mean refractive index of 1.25). Baseline correction and normalisation to the highest peak were carried out. This allows reliable comparison of data despite possible differences regarding sample consistency.

### Lithic use wear analysis

Lithic use-wear analysis plays a fundamental role in determining the techniques employed in the processing of animal carcasses within subsistence behaviour. Use-wear analysis takes into account the fact that repeated actions performed with a stone tool leave macroscopic and microscopic evidence of friction^26–35^. By examining the surfaces and used edges of 13 replicated stone tools both macroscopically (impact edge damage, such as scars and fractures) and microscopically (edge rounding, polish, striation) this study aims to identify and associate use-wear to specific butchery actions including bone fracturing, disarticulation and defleshing and compare to use-wear generated by processing other animal species, to distinguish tortoises and freshwater turtles butchery from other faunal activities. For use-wear research, the spectrum of published experimental butchery work covers a wide array of fauna and use-wear obtained is well characterised, with exception of chelonid species^31,36^. Results presented here are, therefore, particularly useful for further lithic use-wear studies.

The use-wear study of 13 replicated tools (6 quartzite hammerstones and 7 flakes made of flint) was conducted at LARC-Archaeosciences Laboratory, Património Cultural, I.P. (Lisbon, Portugal), where optical and digital microscopy is available. Prior to the experiments, the edges and surfaces of the replicated stone tools were photographed using the digital microscope Hirox HR 5000E to monitor the modification of the original edges and, therefore, to be able to compare the edges morphology prior and after the experiment.

Examination of the edges and surfaces of the stone tools used in experiments followed the established protocol in use-wear analysis consisting in careful cleaning with alcohol to remove any grease and residues it may have contained that could adhere to it and documentation of the tool, followed by a detailed morphological analysis. Subsequently, the tools’ edges and surfaces were examined under low and high magnifications by combining optical bright field reflected light and digital microscopy. Stone tools were first examined using an Olympus SZX12 and the Hirox HR 5000E, to characterise macroscopically the used zones (such as presence of impact scars, edge rounding). Edges and surfaces were then analysed at high magnification (200X) with a Microscope Olympus BX60 for the observation and identification of the microscopic use-wear features, such as polishes indicative of specific actions performed.

## Results

### Processing movements

#### Cooked animals

**Fig. 1:**
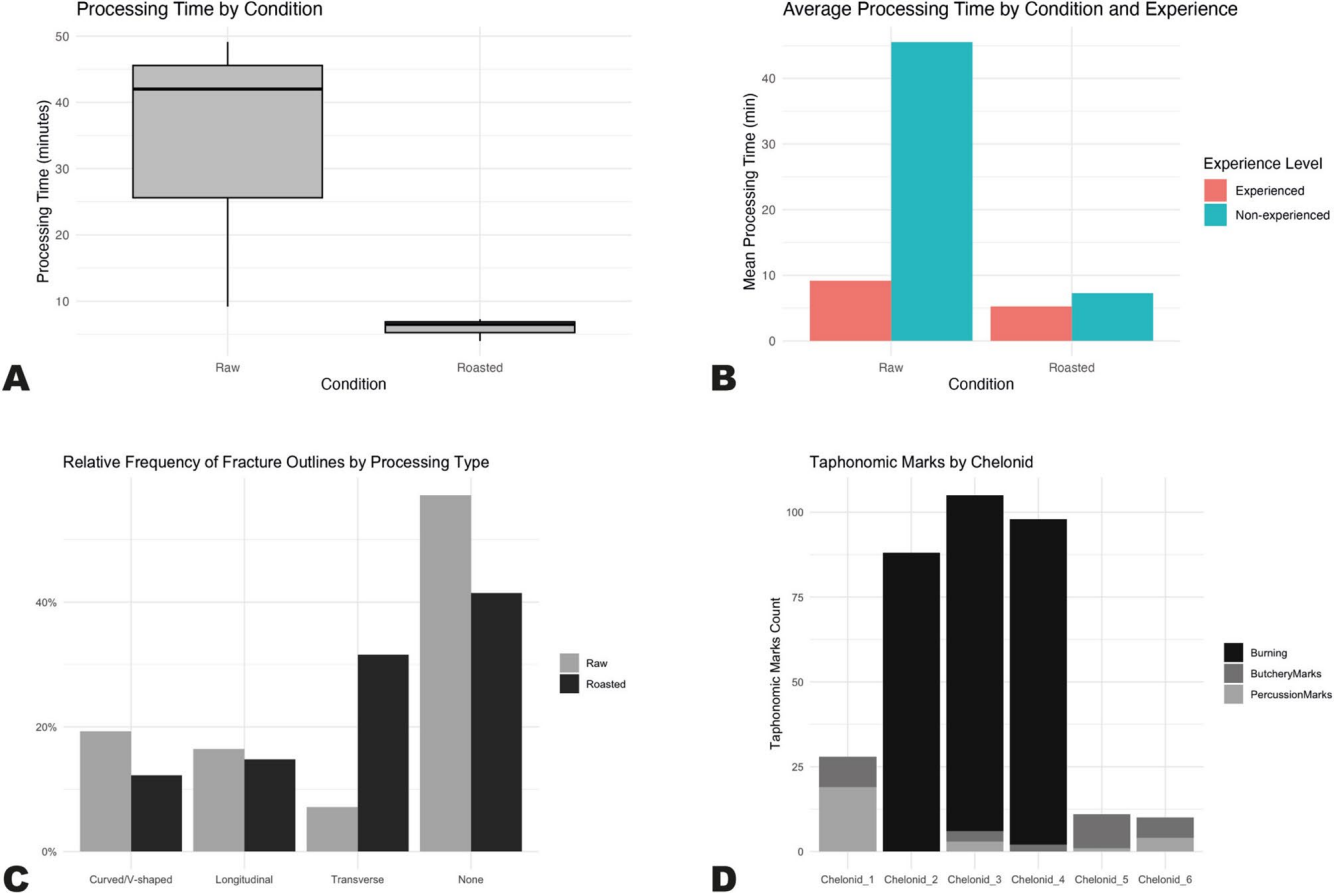
**(A)** Boxplot comparing processing times between raw and roasted chelonid specimens, showing significantly shorter times for roasted individuals. **(B)** Mean processing time by condition and experience level, indicating that while experience reduces processing time for raw specimens, both experienced and non-experienced individuals achieve similarly low times when processing roasted animals. These results highlight the role of heat in reducing labour intensity and levelling skill disparities. **(C)** Relative frequency of fracture outline types by processing method, with raw specimens exhibiting more curved and longitudinal fractures, and roasted specimens more frequently showing transverse and unfractured (none) outlines. **(D)** Total taphonomic marks by specimen, with raw-processed chelonids displaying more percussion and butchery marks, while roasted individuals are characterised by high frequencies of burning.

Chelonids 2, 3 and 4 were cooked prior to butchering (Tab. 1; SI 1; SI 2; https://dataverse.csuc.cat/dataset.xhtml?persistentId=doi%3A10.34810%2Fdata2413). To separate the carapace from the plastron, the shell was struck laterally at the bridge area, where the plastron and carapace are joined. In all roasted specimens, a hammerstone was employed and, after a couple of strikes, the bridge was successfully broken. Additionally, the carapace fractured transversally, which facilitated further disarticulation but rendered it unsuitable for potential secondary uses, such as serving as a bowl or container. The carapace and plastron were then easily separated by cutting the cooked internal skin and meat with flint flakes. Evisceration was performed manually without difficulty. The forelimbs, hindlimbs, heads and tails were removed with minimal effort by manual rotation, pulling, or flexing, and in the case of Chelonid 4, occasionally assisted by simple cuts with flake TT2. The butchering process for these cooked specimens ranged from 5 to 7 min (Fig. 1A, 1B) and was completed with ease by both experienced and non-experienced operators.

#### Raw animals

Chelonids 1, 5 and 6 were processed in their raw state (Tab. 1; SI 1; SI 2; https://dataverse.csuc.cat/dataset.xhtml?persistentId=doi%3A10.34810%2Fdata2413). The carapace was struck laterally at the hinge area. This task was performed using quartzite hammerstones, which left distinct macroscopic and microscopic marks on their surfaces (Fig. 5D). Separating the carapace from the plastron presented significant challenges due to the rigidity of the bridges that join the chelonids’ shell. Multiple hammerstone strikes were required to break the shell, with clear indications that a more experienced operator would achieve this task with fewer blows compared to someone lacking experience (Figs. 1A, 1B). The internal skin of the chelonids was particularly tough and adhered strongly to the bones of the carapace, making it difficult to cut using a flake. During the processing of Chelonids 1 and 6, the distal end of the flint flakes broke due to the resistance of the tissue. For Chelonid 5, the edge of flint flake TT4 was extensively employed for cutting bone and tissue during the separation of the carapace from the shell (S.I. 4). The intensive use of TT4 demanded the subsequent deployment of an additional flint flake, TT3 (Fig. 5A), which offered a sharper edge, thereby ensuring the continuation of efficient cutting performance. Fully separating the carapace from the plastron in their raw state required significant physical effort.

Once the carapace was opened, the internal viscera were manually removed with relative ease for Chelonids 1 and 6. The viscera removal for Chelonid 5 required additional assistance using the flint flake TT3. The subsequent step involved the disarticulation of the appendicular skeleton. This process was achieved by manually rotating and overextending each limb, supplemented by transversal cuts at the joint areas connecting the legs to the carapace. The heads and tails were removed using the same method, involving manual handling and transversal cuts at the respective joints to ensure complete separation. The entire butchering process ranged between 9 and 49 min (Fig. 1A, 1B). Given that Chelonids 1 and 5 were processed by non-experienced operators (Tab. 1), while Chelonid 6 was processed by an experienced operator, the marked differences in processing times, despite all animals being of similar size, highlights the significant impact of skill and familiarity on the efficiency of the butchering technique.

### Skeletal part representation

The skeletal part representation across the analysed chelonid specimens (Chelonids 1–6) indicates that most anatomical elements are present, regardless of whether the animals were processed raw or cooked (https://dataverse.csuc.cat/dataset.xhtml?persistentId=doi%3A10.34810%2Fdata2412). This reflects the overall effectiveness of the butchering procedures in recovering most major skeletal components.

**Fig. 2:**
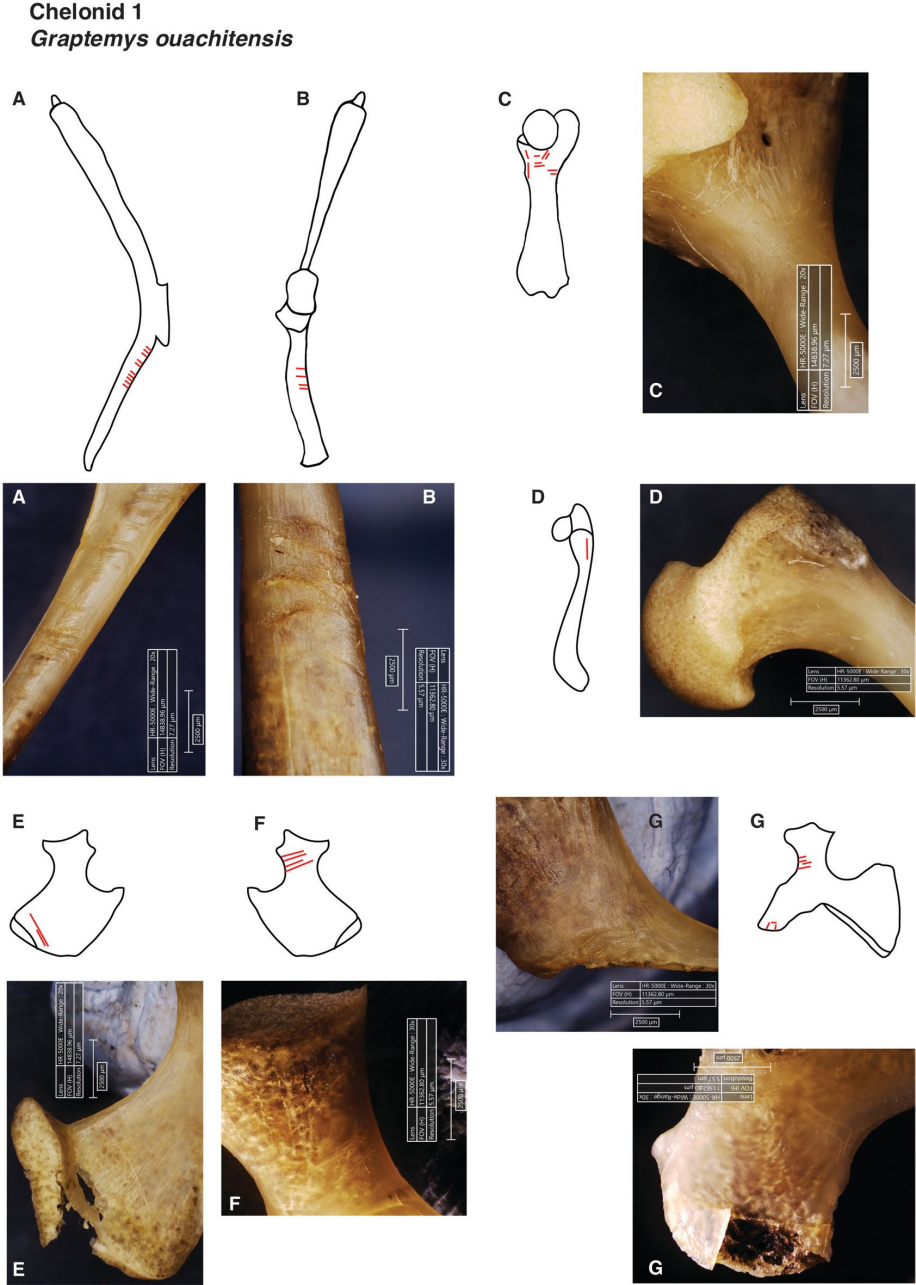
Examples of cuts on the appendicular skeleton of *Graptemys ouachitensis* (Chelonid 1) after being experimentally processed uncooked by a non-experienced person. Illustrations are not drawn to scale and should be interpreted as conceptual sketches; they are not suitable for species identification. **(A)** Left scapula, cranial side. (**B)** Right scapula, lateral side. **(C)** Left humerus, dorsal side. **(D**) Right humerus, medial side. **(E)** Left ischium, dorsal side. **(F)** Right ischium, dorsal side. **(G)** Right pubis, dorsal side.

**Fig. 3:**
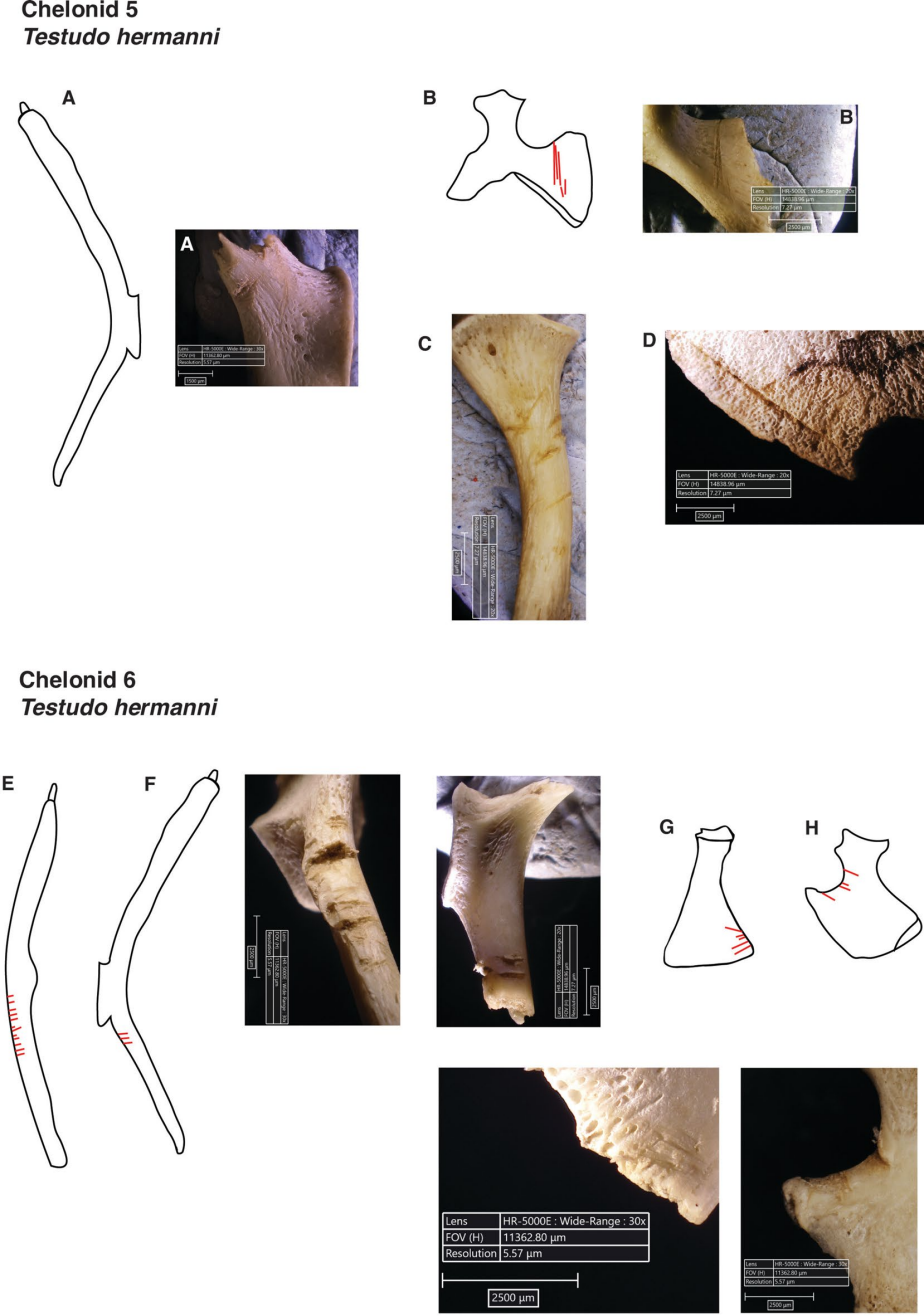
Examples of cuts on *Testudo hermanni* (Chelonids 5 and 6) after being experimentally processed uncooked by an experienced person. Illustrations are not drawn to scale and should be interpreted as conceptual sketches; they are not suitable for species identification. **(A)** Left scapula, cranial side. **(B)** Right pubis, dorsal side. **(C)** Right ilium, lateral side. **(D)** Right hyoplastron, lateral side. **(E)** Left scapula, medial side. **(F)** Right scapula, lateral side. **(G)** Right coracoid, dorsal side. **(H)** Right ischium, dorsal side.

Among the raw-processed individuals (Chelonids 1, 5 and 6), representation includes all axial and appendicular elements. Long bones are consistently documented, along with fragmented parts of the carapace and plastron. This representation suggests that although raw processing posed greater challenges – evidenced by fragmentation and longer processing times – the integrity of long bones was generally preserved (Figs. 2, 3).

The cooked specimens (Chelonids 2, 3 and 4) similarly display a well-distributed skeletal representation, with most long bones recovered in complete condition. The application of heat significantly eased disarticulation and resulted in minimal breakage of large skeletal elements. However, an exception was observed in the frequent absence of smaller anatomical parts, particularly phalanges. These diminutive bones appear to have been more susceptible to thermal damage or loss during roasting, likely due to their size and lower resistance to heat exposure. As such, their underrepresentation in the cooked assemblages may be attributed to post-roasting fragmentation and dispersal.

### Bone surface modifications

Overall, raw-processed specimens exhibit a greater proportion of complete bones, while roasted specimens are more highly fragmented. Raw chelonid individuals present a higher incidence of curved/V-shaped and longitudinal fractures, whereas transverse fractures were more commonly observed in roasted chelonids. (Fig. 1C).

**Fig. 4:**
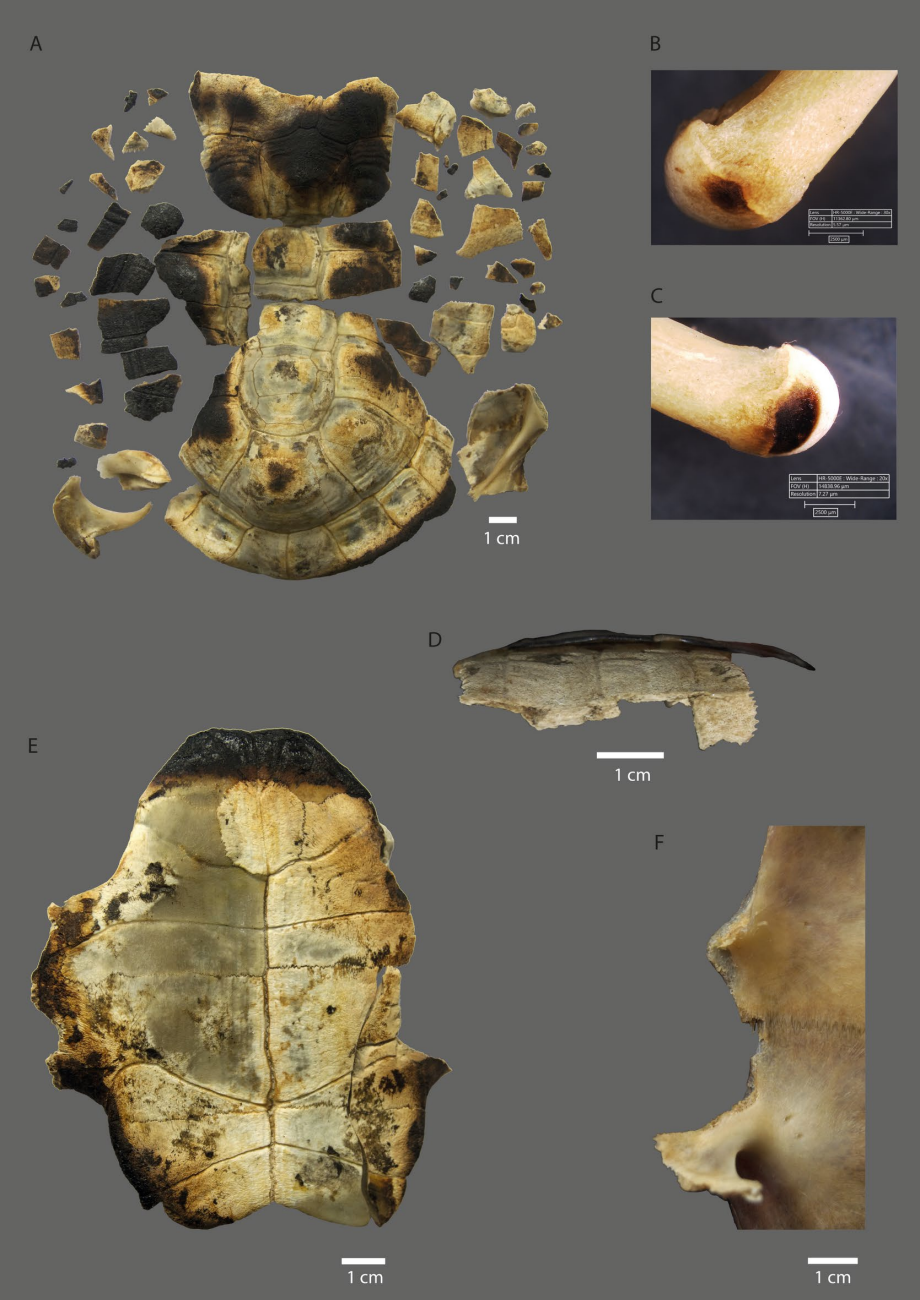
Evidence of burning and percussion on chelonid specimens. **(A)** Dorsal view of *Testudo hermanni* (Chelonid 3) carapace with black burning marks. **(B)** Left humerus of *Testudo hermanni* (Chelonid 4) showing a black burning mark under Hirox microscope. **(C)** Right humerus of *Testudo hermanni* (Chelonid 4) with black burning mark under Hirox microscope. **(D)** Right peripherals plates 4, 5, and 6 of *Testudo hermanni* (Chelonid 5) with lateral percussion notches. **(E)** Exterior plastron of *Testudo hermanni* (Chelonid 4) with black burning marks. **(F)** Interior plastron of *Graptemys ouachitensis* (Chelonid 1) showing percussion notches.

The roasted individuals (Chelonids 2, 3 and 4) predominantly exhibited burning marks, with minimal evidence of tool use (Fig. 1D; Fig. 4; Tab. 2; S.I. 3). All front and hind phalanges from these three specimens were either burnt or absent, most likely having disintegrated during roasting as a result of direct contact with the hot embers. Only five long bones exhibit burning marks: three humeri (Fig. 4B, 4 C), one femur and one tibia. All are complete specimens, with black burns on the cranial surface of their distal ends. The remaining burnt specimens (n = 270) originate predominantly from the carapace (n = 238), with a smaller number identified on plastron plates (n = 32). All carapace shells show signs of burning with clear propensity for heat-induced black alterations on peripheral (n = 83) and costal (n = 75) plate fragments. Similarly, brown and black burns can be observed on all plastron elements, but they are mainly located on the exterior and lateral edges of the bones (Fig. 4A, 4E; S.I. 3).

**Table 2:** Bone surface modifications of the six chelonid specimens following the experimental processing. In the table, the term “None” refers to bone elements that exhibited no visible surface modifications. Abbreviations: Ext = exterior surface; Int = interior surface.

	CHELONID 1 (raw)		CHELONID 2 (roasted)		CHELONID 3 (roasted)			CHELONID 4 (roasted)		CHELONID 5 (raw)			CHELONID 6 (raw)	
PERCUSSION														
Pit	Peripheral	1		0		Femur shaft Tibia shaft	1 1		0			0		0
Notch	Peripheral ext Hyoplastron ext Hypoplastron ext	1 1 1		0		Hypoplastron ext	1		0		Hyoplastron ext	1	Peripheral ext Hyoplastron ext Hypoplastron ext	2 1 1
Impact flake	Peripheral ext Hypoplastron Plastron ind ext	11 1 2		0			0		0			0		0
Fissure	Xiphiplastron ext	1		0			0		0			0		0
None		43		106			118		117			38		35
% Percussion		30%		0%			2.5%		0%			2.6%		10.3%
INCISION														
Cut	Scapula procoracoid Coracoid shaft Humerus proximal Ischium shaft Ischium proximal Pubis proximal Pubis distal	2 1 2 1 1 1 1		0 0 0 0 0		Humerus proximal Scapula procoracoid	1 2	Scapula glenoid Pubis proximal	1 1		Scapula glenoid Scapula shaft Ilium shaft Ischium shaft Pubis shaft Femur shaft Hyoplastron ext	1 1 2 1 2 2 1	Scapula procoracoid Radius distal Ischium distal Pubis distal Femur distal	2 1 1 1 1
None		53		106			118		115			29		33
% Cut		14.5%		0%			2.5%		1.7%			25.6%		15.4%
BURNING														
Brown		0	Nuccal edge Neural ext Costal ext Peripheral ext Peripheral edge Suprapygal extl	1 1 14 2 8 1		Neural ext Costal ext Peripheral ext Peripheral edge Pygal edge Carapace ind ext Hyoplastron edge Xiphiplastron Tibia distal	5 5 16 2 1 3 1 1 1	Neural ext Costal ext Peripheral ext Entoplastron ext Hyoplastron ext Hypoplastron ext	5 3 8 1 2 2			0		0
Brown-Black		0	Peripheral ext Peripheral edge Pygal edge Epiplastron ext Hyoplastron ext	1 8 1 1 1		Neural ext Costal ext Peripheral ext Suprapygal ext	1 12 2 1	Nuccal ext Neural ext Costal ext Peripheral ext Peripheral edge Pygal ext Carapace ind ext Hypoplastron edge Xiphiplastron edge	1 1 9 15 1 1 1 2 2			0		0
Black		0	Neural ext-int Costal ext Costal ext-int Peripheral ext Carapace ind ext Carapace ind ext-int Humerus distal Femur distal Epiplastron ext Hyoplastron ext Hypoplastron ext Xiphiplastron ext Xiphiplastron edge Plastron ind ext Plastron ind ext-int	3 1 8 3 12 8 1 1 1 1 2 1 1 5 1		Nuccal ext Neural ext Costal ext Peripheral ext Peripheral ext-int Carapace ind ext Epiplastron edge Hyoplastron edge Hypoplastron edge Xiphiplastron edge	1 1 17 4 6 10 2 1 1 1	Neural ext Costal ext Peripheral ext Peripheral edge Carapace ind ext Humerus distal Epiplastron edge	2 6 3 4 19 2 2			0		0
None		62		18			26		25			39		39
% Burning		0%		83%			78.5%		78.6%			0%		0%

Raw-processed specimens (Chelonids 1, 5 and 6) presented higher frequencies of butchery and percussion marks (Figs. 1D, 2, 3, S.I. 3). A total of 30 skeletal elements exhibited cut marks (Tab. 2; Fig. 2; Fig. 3), primarily located on appendicular elements, particularly girdle bones such as the scapula (n = 9, from which 6 are located on the procoracoid), and pelvic elements including the pubis (n = 6) and ischium (n = 4). Cut marks were identified on the hyoplastron in only one individual (Chelonid 5; Fig. 3), where the mark was associated with a percussion notch on the lateral side of the element. Most bones exhibiting cut marks tend to show multiple incisions, with only 30% (n = 9) displaying a single, isolated cut. All cuts have a straight delineation and 67% (n = 20) are transverse to the axis of the bone; only three bones show longitudinal cuts.

A total of 27 bones exhibit percussion marks (Tab. 2; Fig. 4; S.I. 3). Of these, more than half (56%, n = 15) are located on the peripheral plates of the carapace or on the lateral sides of plastron bones (37%, n = 10), predominantly in uncooked specimens. Most of these modifications correspond to impact flakes (52%, n = 14) and notches (33%, n = 9) (S.I. 3). Only three bones belonging to a roasted individual (Chelonid 3) display percussion marks: one notch situated on the lateral side of the hypoplastron, and two pits – one on the medial surface of a femoral shaft and another on the medial side of a tibial shaft (https://dataverse.csuc.cat/dataset.xhtml?persistentId=doi%3A10.34810%2Fdata2412).

### FTIR

**Fig. 5:**
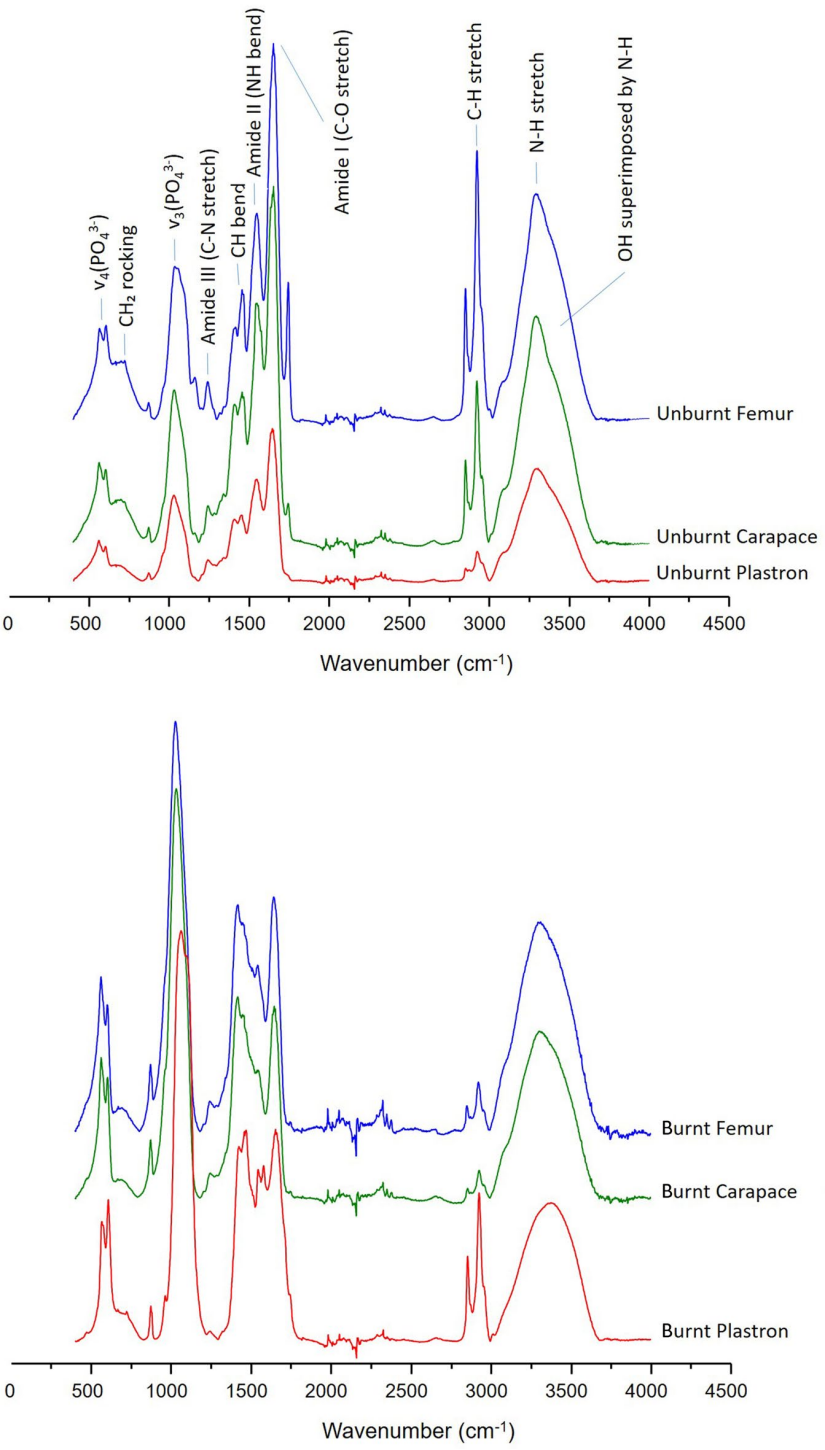
Examples of FTIR-ATR spectra of unburnt and burnt femur, carapace and plastron of experimentally processed chelonids. Heat exposure resulted in the loss of some organic matter (e.g. Amides I, II and III) while the phosphate content (~ 560 cm^−1^, ~ 600 cm^−1^ and ~ 1030 cm^−1^) became more prominent.

The infrared spectra of plastron and carapace samples taken from uncooked specimens appeared to show a mix of bone and β-keratin characteristics (Fig. 5). The characteristic phosphate bands of bone were noticeable at wavenumbers ~ 560 cm^−1^, ~ 600 cm^−1^ and ~ 1030 cm^−1^^37,38^. As for β-keratin, the collagen proxies corresponding to amide I (~ 1650 cm^−1^), amide II (~ 1540 cm^−1^) and amide III (~ 1240 cm^−1^) were the most noticeable features^39^ although these were also expected to have some contribution from bone itself. The same happened for the N–H stretch at ~ 3287 cm^−1^ and the C-H stretch around the 2900 cm^−1^ region which are also typical of the molecular profiles of both non-defatted bone and β-keratin^40–43^. In turn, the CH_2_ rocking at ~ 720 cm^−1^ (long-chain lipid tails) appeared to be typical of β-keratin^40,42,43^.

After a short cooking procedure, infrared spectra of roasted chelonids showed an important reduction of bands associated to organic matter (amides, lipids) compared to the phosphate peaks which became more sharpened. However, as is expected from a cooking procedure, the heat exposure was not strong enough to eliminate the organic matter. It should be noted that the heat-induced changes observed in the infrared spectra can also be caused by diagenesis^44^ preventing the clear identification of low- and medium-intensity heat exposures in bone^45^. No unmistakable indications of heat exposure could be found in the spectra because typical high-temperature features were not present – e.g. OH liberation at ~ 630 cm^−1^,OH stretching at ~ 3572 cm^−1^^46^.

One surprising find was that the infrared spectra obtained from femurs, which reportedly do not have any keratin contribution, showed molecular profiles very similar to the plastron and carapace. In particular, the very high intensity of amides was unusual when compared with non-defatted mammal bone^42^. A skin β-keratin^47^ contamination of femoral samples may have occurred. This may also be partly explained by potentially high amounts of collagen in chelonids compared to mammal bones and/or by the relatively young age of the specimens used in this study, making them more prone to have higher collagen contents.

Manganese-induced stains can sometimes be mistaken for charred bone. However, the vibrational modes of manganese oxide are not easily identified through FTIR analysis^48^ since they tend to overlap with those from bones – e.g. phosphates, carbonates, amides^49^. Therefore, FTIR appears to have little potential to discriminate cooking procedures of archaeological chelonid remains.

### Lithic use-wear

**Fig. 6:**
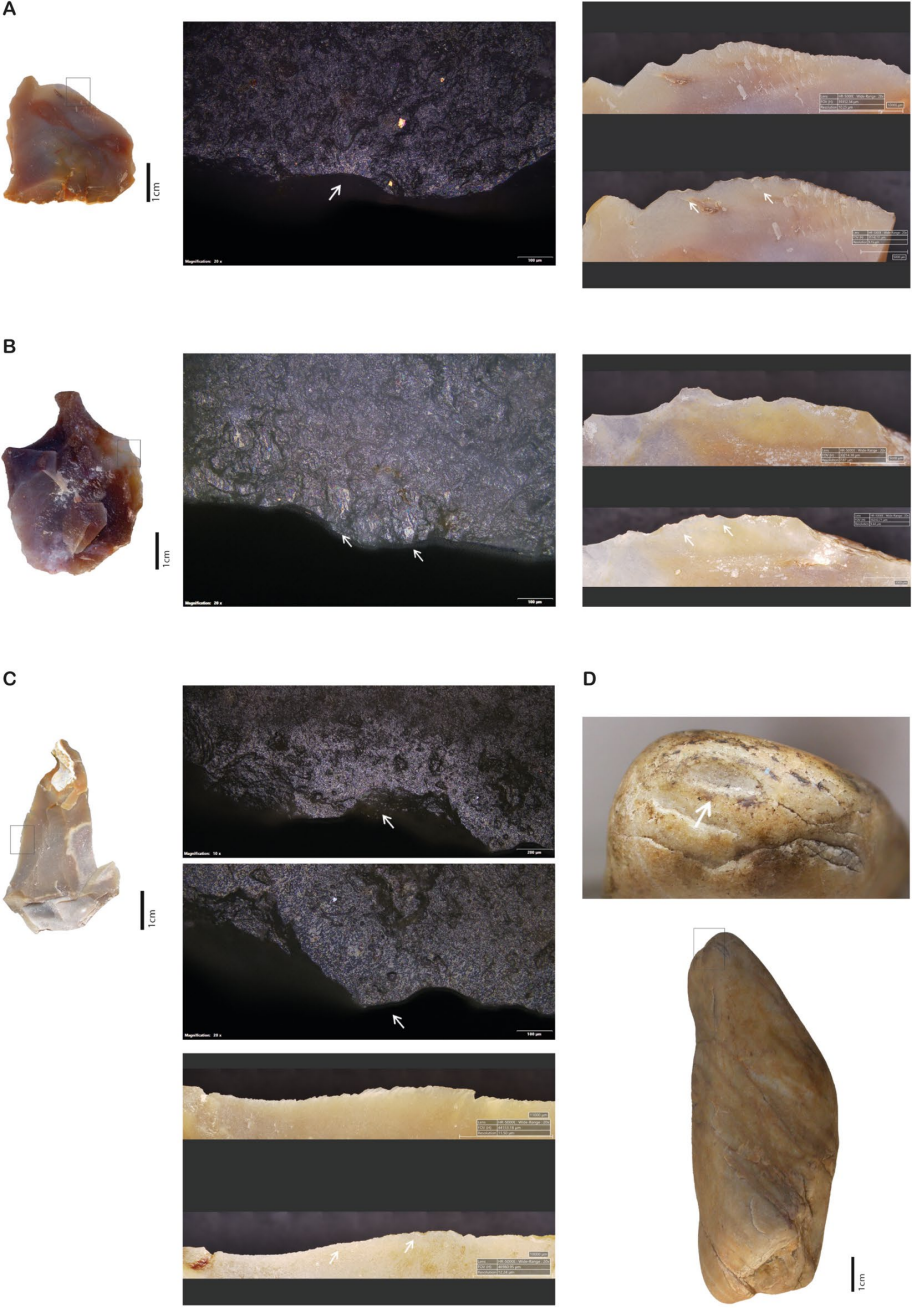
Lithic use-wear from butchering raw Chelonids. **(A)** Flint flake TT3 (Chelonid 5): flake overview (left); disarticulation polish at 200 × (middle); edge before (top) and after (bottom) use under Hirox microscope, with white arrows marking macroscopic edge changes (right). **(B)** Flint flake TT1 (Chelonid 6): flake overview (left); polish from cutting bone and tissue at 200 × (middle); edge comparison before and after use with edge modification (right). **(C)** Flint flake 6 (Chelonid 1): flake overview (left); disarticulation polish at 200 × (right); edge comparison before and after use with visible modifications (bottom left). **(D)** Quartzite hammerstone TT6 (Chelonid 5): impact scar on distal end from use.

Among the 13 pieces examined – 6 quartzite hammerstones and 7 flakes made of flint – only 4 flakes exhibit characteristic microscopic use-wear in the form of polishes (TT1, TT4, TT5 and Flake 6; Fig. 6 and S.I. 4). While only small patches of polish with a reticular shape and compact appearance sparsely distributed along the edge can be observed on most of these tools, Flake 6 shows microscopic polish particularly well developed (Fig. 6C). Whether this could be related with the processed specimen features of the species itself is something that requires further explanation.

Stone tools results show no difference in the appearance of microscopic use-wear depending on whether the chelonids were processed raw or cooked, but this observation needs further testing since it is very likely that it could also be related to the short duration of use of the tools, preventing the development of microscopic polishes.

Regarding the quartzite hammerstones, only the artefact TT6 bears recognisable impact scars from percussion formed during the carapace removal of Chelonid 5 (Fig. 6D).

The set and features of macroscopic and microscopic traces of use-wear observed on flakes used to disarticulate and defleshing are similar to those obtained in the context of butchery of other animals^27,28,31,34–36^. Use-wear features observed include the presence of scars located on both sides of the cutting edge associated to patches of compact polish with a reticular shape distributed along the edge.

Particularly noteworthy is the macroscopic modification of the used edges original outline, in the form of loss of raw material and the presence of numerous scars on both sides of the used edge, well visible on photographs of cutting edges prior and after use made with digital microscope Hirox HR500E. The edge removals (Fig. 5A, B, C; S.I. 4) result from the contact between the flake and the fibrous textures of muscle tissue but occasionally touching more resistant material, such as bone or cartilage. Yet, while macroscopic modification of the original edges of flakes is well visible, microscopic use-wear traces such as polishes are globally more tenuous, which is consistent to the working of butchery on small game and over a short time duration.

## Discussion

Ethnographic evidence highlights the multifaceted role of chelonids in human subsistence strategies. Across cultures and time periods, tortoises, freshwater and marine turtles have served as valuable resources not only for their meat, viscera and fat, but also for their eggs, with deep cultural associations evident in different communities. In the Middle Orinoco region of South America, the *Podocnemis expansa* (Arrau turtle) has been a critical resource for Indigenous people since precolonial times. Practices such as fishing and egg collection remain integral to their cultural identity and subsistence activities^50^. In Mexico, the Maya people of Tabasco continue to associate turtle consumption with social identity and cultural heritage, despite the ecological pressures impacting these practices^51^. Australian Aboriginal communities also highlight the cultural importance of sea turtles, integrating them into trade, storytelling and dietary traditions, showcasing a deep and enduring relationship^52^. Meanwhile, in Bali communities that once relied on turtles for consumption, trade and rituals have transitioned towards conservation-focused practices, adapting their rituals and integrating hatchling-release initiatives^53,54^.

Regardless of whether chelonids were processed for consumption or other uses, understanding how efficiently they can be processed is crucial to interpreting their role in subsistence and cultural practices. Our experimental results clearly show that cooking significantly enhances processing efficiency. Raw-processed specimens required considerable physical effort and longer processing times (up to 49 min), with experienced operators outperforming novices. The rigidity of the shell and internal tissues posed significant challenges, often leading to tool damage and prolonged disarticulation efforts. In contrast, cooked specimens were processed in only 5–7 min, regardless of experience level. Heat application softened connective tissues, facilitated disarticulation and reduced the need for tool use for cutting or disarticulating. However, roasting led to complete fracturing of the carapace, preventing any potential reuse as containers.

Other surface modifications further support these finds. Raw-processed chelonids exhibit a higher proportion of butchery and percussion marks, particularly on appendicular elements. Cut marks were most frequent on gridle elements, while percussion marks appeared on the hinge areas of carapace and plastron plates, consistent with the effort required to separate these structures. Conversely, roasted specimens showed minimal tool marks but high frequencies of burning, especially on peripheral and costal plates of the carapace. Smaller elements, like phalanges, were often missing, likely due to thermal degradation during roasting.

These experimental insights provide a robust framework to interpret archaeological finds. Research across key Palaeolithic sites in the Iberian Peninsula and the Mediterranean Basin reveal a structured and consistent approach to tortoise exploitation, characterised by in-shell roasting and precise anatomically disarticulation. Burning traces are predominantly localised on the dorsal carapace – particularly the costal and peripheral plates – indicating deliberate thermal processing (e.g.^10–13,20^). At Bolomor Cave, over 63% of *Testudo hermanni* remains show such dorsal burning, accompanied by human tooth marks and cutmarks^20^. Similar concentrations of burnt remains occur at Kebara Cave, Qesem Cave, Gruta da Oliveira and Gruta da Figueira Brava, with the most intense burning observed in the carapace and minimal ventral exposure, suggesting controlled roasting practices^10–12^. These thermal alterations are often accompanied by fragmentation, percussion marks and disarticulation focused on the hinge area, as evidenced for example, at Hayonim Cave^13^, Gruta da Oliveira and Gruta da Figueira Brava^11^. At Mealhada, although more fragmentary, chelonid remains (including Testudinidae, *Mauremys leprosa* and *Emys orbicularis*) exhibit blackening and thermal fractures, further suggesting fire exposure, likely from roasting or hearth deposition^21^. However, our experimental results confirmed the little potential of FTIR analysis on the identification of heat exposure related with cooking.

Butchery evidence reinforces this narrative. At Sima del Elefante (~ 1.29 Ma), *Testudo hermanni* remains present incisions on neural and costal plates^2^. At Bolomor, cutmarks near limb joints and percussion notches on plastron plates indicate post-roasting processing^20^. At Kebara, cutmarks on the bridge and anterior plastron hinge – such as the one found in Chelonid 5 – suggest efforts to sever soft tissue, consistent with targeted visceral access and implying sequential disarticulation^10,12^.

Experimental butchery involving chelonid remains is still rarely documented in lithic use-wear research. The study presented here helps address this gap by offering a comparative framework to interpret archaeological use-wear patterns. This approach contributes to identifying specific butchery techniques used on small game and provides key insights into early technological practices and dietary behaviours. By replicating the processing of chelonids – such as shell breaking and disarticulation using stone tools – we were able to document distinct wear traces, including polishes and edge scars. These traces closely resemble use-wear patterns found on tools associated with the processing of other animal species, both in their appearance and distribution (e.g.,^31,36^).

Our experimental results also contribute to ongoing debates about Middle Palaeolithic subsistence. The incorporation of small game, such as tortoises and freshwater turtles, suggests an adaptive broadening of subsistence strategies that is not fully accounted for by Optimal Foraging Theory, which states that food acquisition decisions are driven by the aim to maximise energy returns while minimising effort and risk^55,56^. These animals, although yielding less meat than large ungulates, are easy to catch due to their predictable behaviours and, in the case of tortoises, for their slow movement (e.g.^57–59^). Archaeological sites like Gruta da Oliveira suggest sustained exploitation over time, including changes in tortoise body size that may reflect harvesting pressure^11^. In this context, Niche Construction Theory may offer a more fitting interpretive framework, highlighting human agency in shaping resource use and environmental interactions (e.g.^60–62^).

From a nutritional standpoint, despite low meat yields, chelonids offer valuable resources. Studies on the Amazonian *Podocnemis expansa* indicate a useful composition of meat, fat and viscera^63^, aligning with our observations. Cooking maximises access to these components with minimal effort, enhancing the caloric return. Moreover, the repeated engagement in chelonid processing likely led to skill acquisition and knowledge transfer, fostering social cohesion within groups.

Archaeological and experimental evidence together point to a shared and culturally transmitted tradition of chelonid exploitation, shaped by a strategic use of fire. The widespread presence of burning, cutmarks and controlled fracturing across geographically diverse archaeological sites indicates that chelonid processing was not random, but rather cognitively sophisticated, culturally embedded and a socially transmitted tradition within Palaeolithic lifeways. The efficiency gains from cooking suggest that early humans may have strategically employed fire not just for dietary enhancement but also for labour reduction. These behaviours align with archaeological finds, such as those at Qesem, where it is evident the central role of fire in dietary practices^16^, and with broader arguments about fire’s role in cognitive evolution and cultural transmission (e.g.^18,64,65^).

Additionally, cooking signals complex cognitive behaviours through preplanning for fire, acquiring suitable materials and coordinating processing tasks, which suggest foresight and strategic thinking. Such behaviours mirror those required in tool production and hunting, reinforcing hypotheses about the role of skill-intensive practices in brain evolution (e.g.^66–69^). Moreover, the ability to process cooked animals equally well by experienced and inexperienced individuals emphasises cooking’s democratising role in food access and group efficiency. By reducing the physical effort and technical skills required to disarticulate and consume animal resources, cooking allows a broader range of individuals – including those with less experience, strength or dexterity – to contribute to and benefit from food processing and consumption. This, in turn, promotes more equitable food sharing and supports inclusive group cohesion.

In conclusion, the integration of chelonids into early human diets was not a random fallback strategy but a sophisticated, planned behaviour shaped by environmental, cognitive and social factors. Cooking emerges as a central improvement that transformed chelonid processing, reduced labour costs, enabled wider participation in food preparation and reinforced group learning.

## Supplementary Information

Supplementary Information 1.

Supplementary Information 2.

Supplementary Information 3.

Supplementary Information 4.

## Data Availability

The datasets generated during and/or analysed during the current study are available in the CORA repository and can be accessed through the following links https:/dataverse.csuc.cat/dataset.xhtml?persistentId = doi%3A10.34810%2Fdata2413 and https:/dataverse.csuc.cat/dataset.xhtml?persistentId = doi%3A10.34810%2Fdata2412 Our study does not involve human participants in the usual sense. All individuals who appear in the images are the authors of the study and no other identifiable persons or patient data are included. For this reason, we consider a formal informed-consent statement from study participants not applicable. All authors explicitly agree to the publication of these images and any associated information in an online open-access format.
