# Supplementary material for: Experimental analysis of roasted and raw turtle butchery and implications for early human cognition and behaviour
Source: Sci Rep. 2025 Dec 24;16:1913. doi: 10.1038/s41598-025-31738-z (PMC12804910; doi:10.1038/s41598-025-31738-z)
Supplement: Supplementary file 1 — Supplementary Information 1. [file 41598_2025_31738_MOESM1_ESM.pdf]

CHELONID 1

Graptemys ouachitensis

LITHIC IMPLEMENTS

Quartzite hammerstone 2a

Flint flake 6

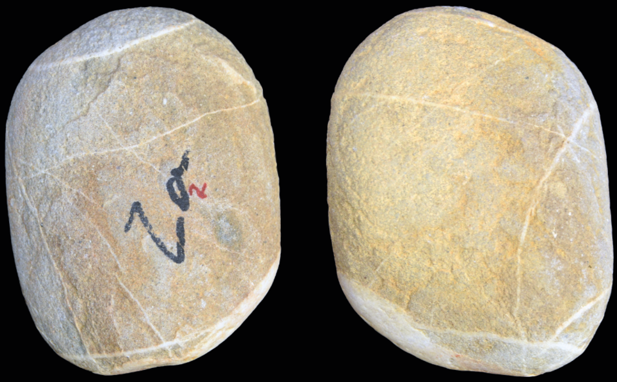

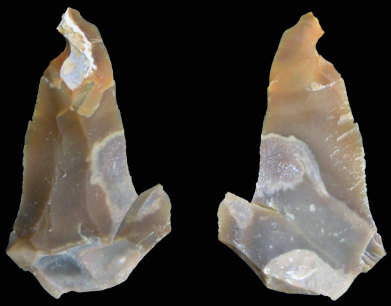

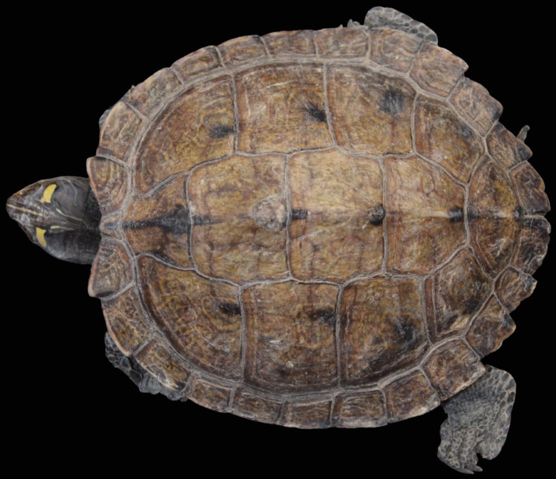

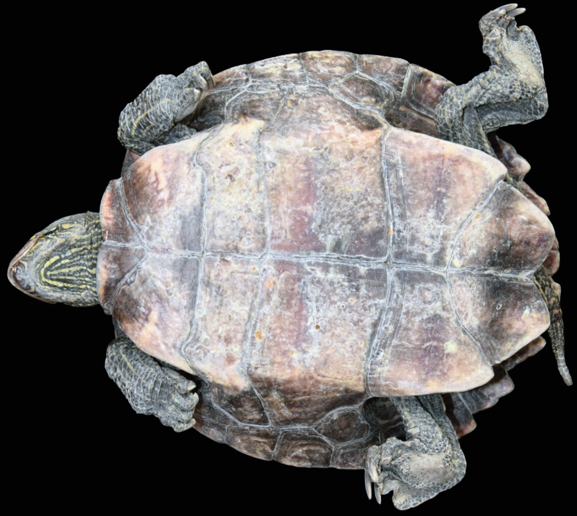

5 cm

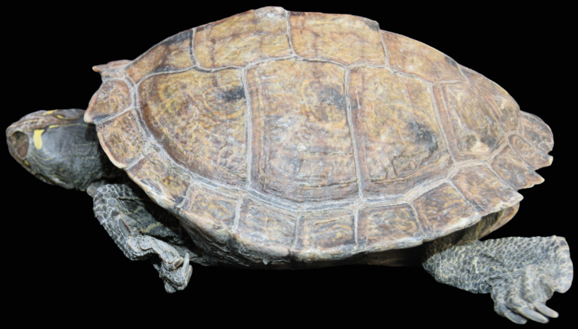

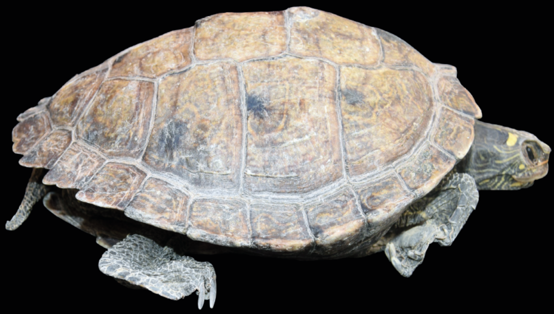

5 cm

CHELONID 2
Mauremys cf. *reevesii*

LITHIC IMPLEMENTS
Quartzite hammerstone 4a
Flint flake 5

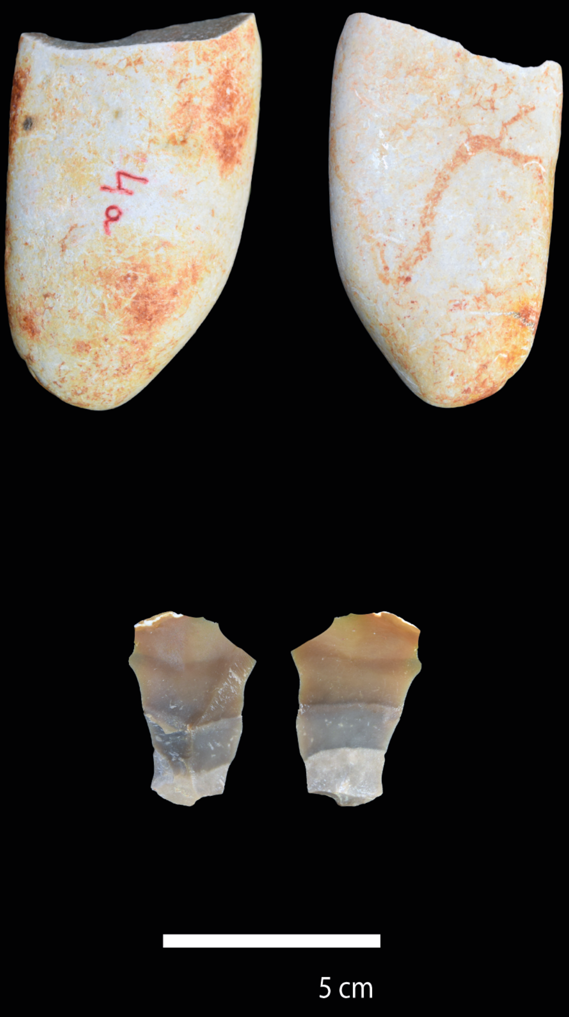

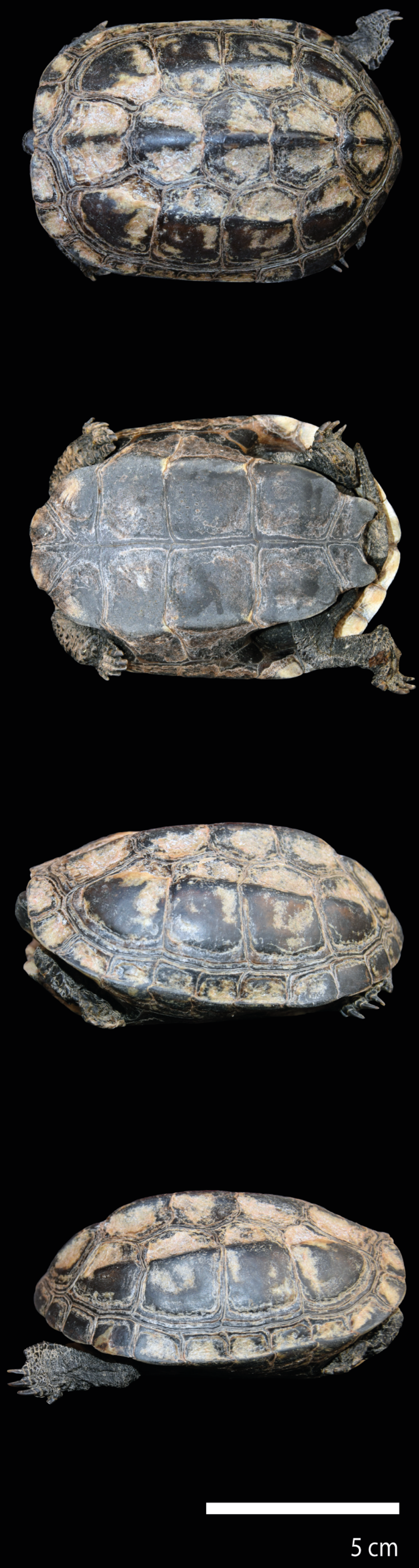

CHELONID 3
Testudo hermanni

LITHIC IMPLEMENTS
Quartzite hammerstone TT9
Flint flake TT5

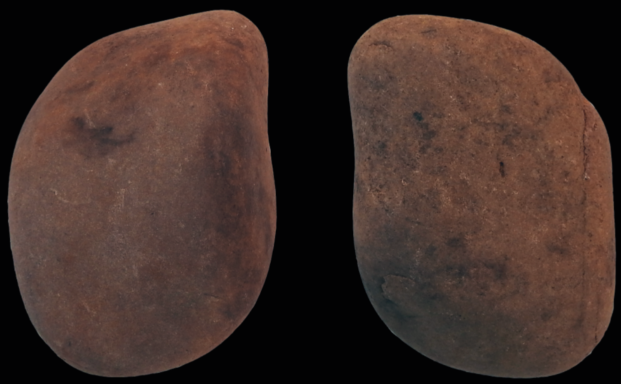

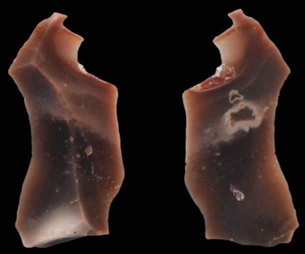

5 cm

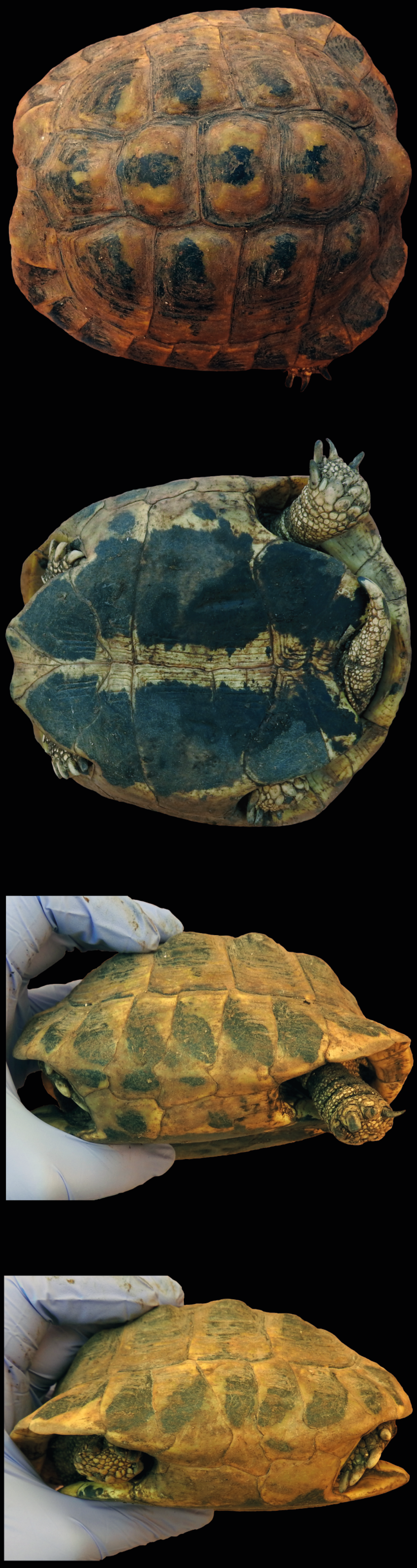

5 cm

CHELONID 4
Testudo hermanni

LITHIC IMPLEMENTS
Quartzite hammerstone TT7
Flint flake TT2

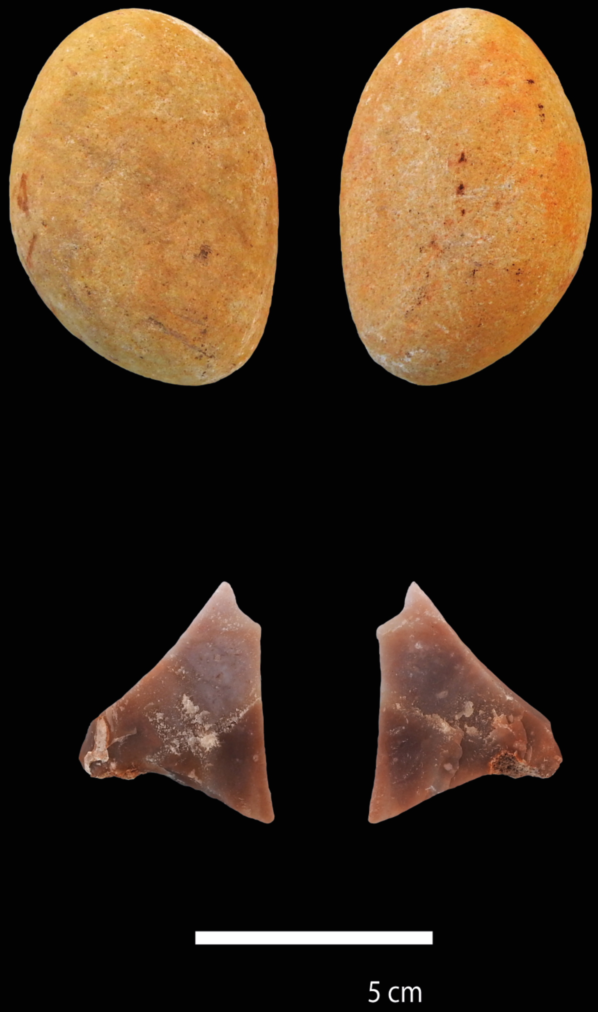

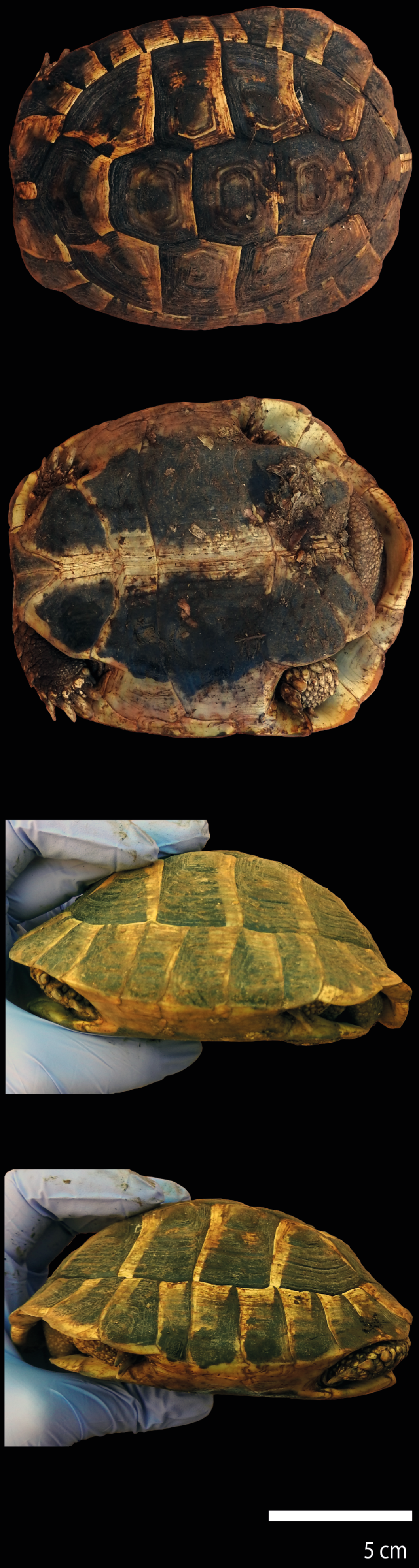

CHELONID 5
Testudo hermanni

LITHIC IMPLEMENTS
Quartzite hammerstone TT6
Flint flakes TT3 and TT4

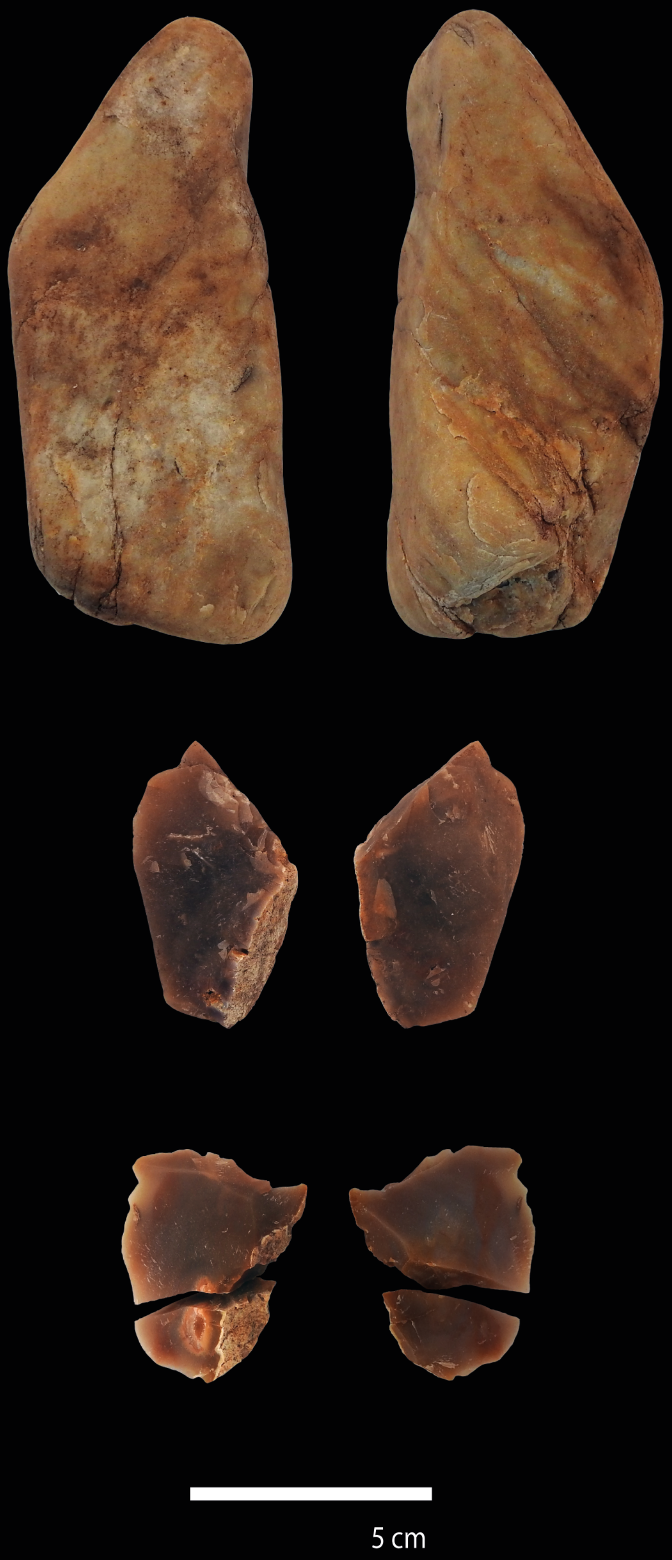

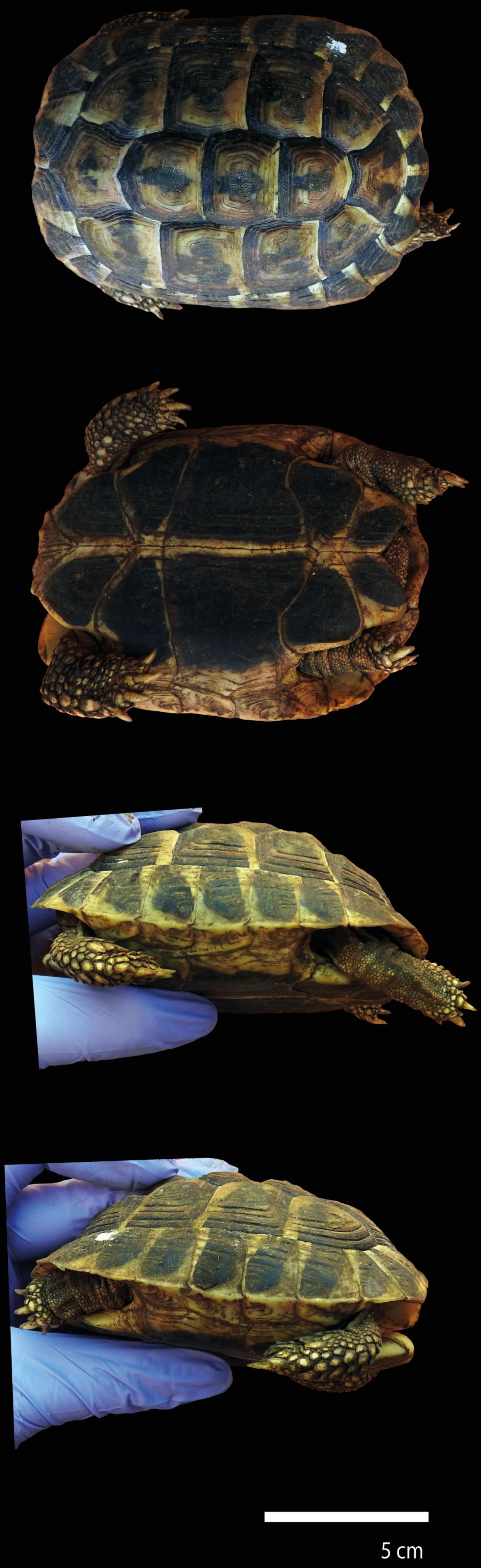

CHELONID 6

Testudo hermanni

LITHIC IMPLEMENTS

Quartzite hammerstone TT8

Flint flake TT1

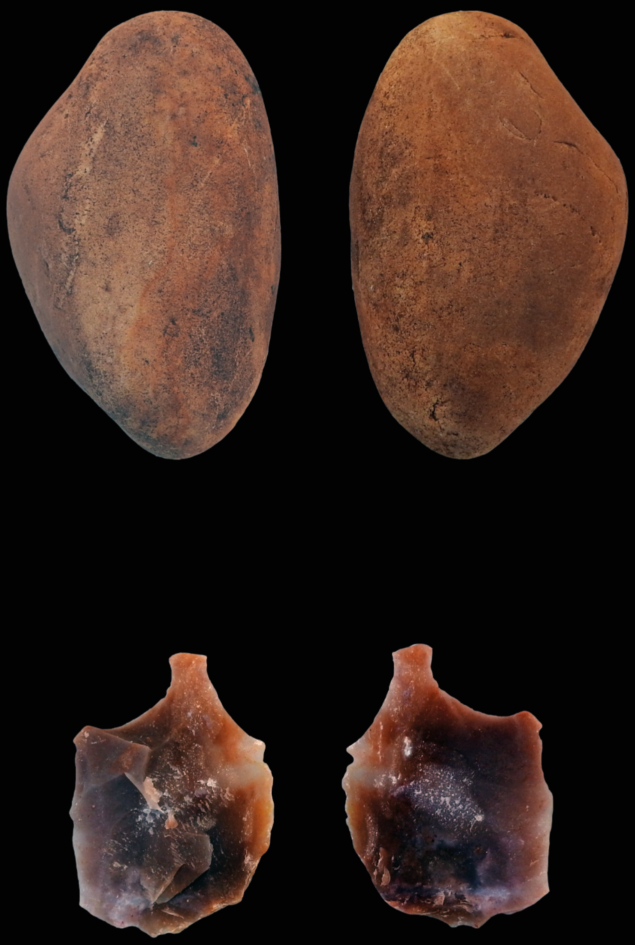

5 cm

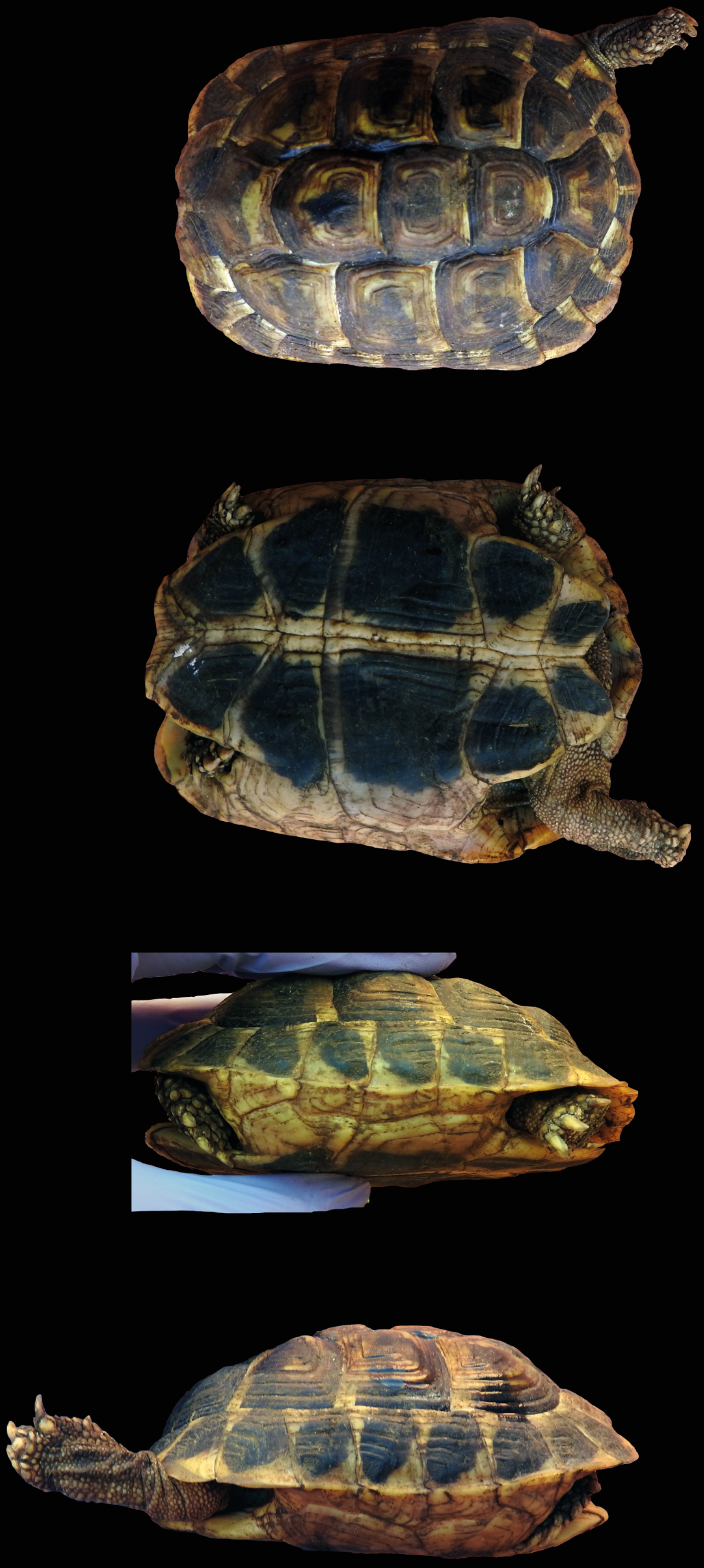

5 cm
