# Supplementary material for: Experimental analysis of roasted and raw turtle butchery and implications for early human cognition and behaviour
Source: Sci Rep. 2025 Dec 24;16:1913. doi: 10.1038/s41598-025-31738-z (PMC12804910; doi:10.1038/s41598-025-31738-z)

Weighing, measuring and photographing:
Chelonid specimens and lithic implements

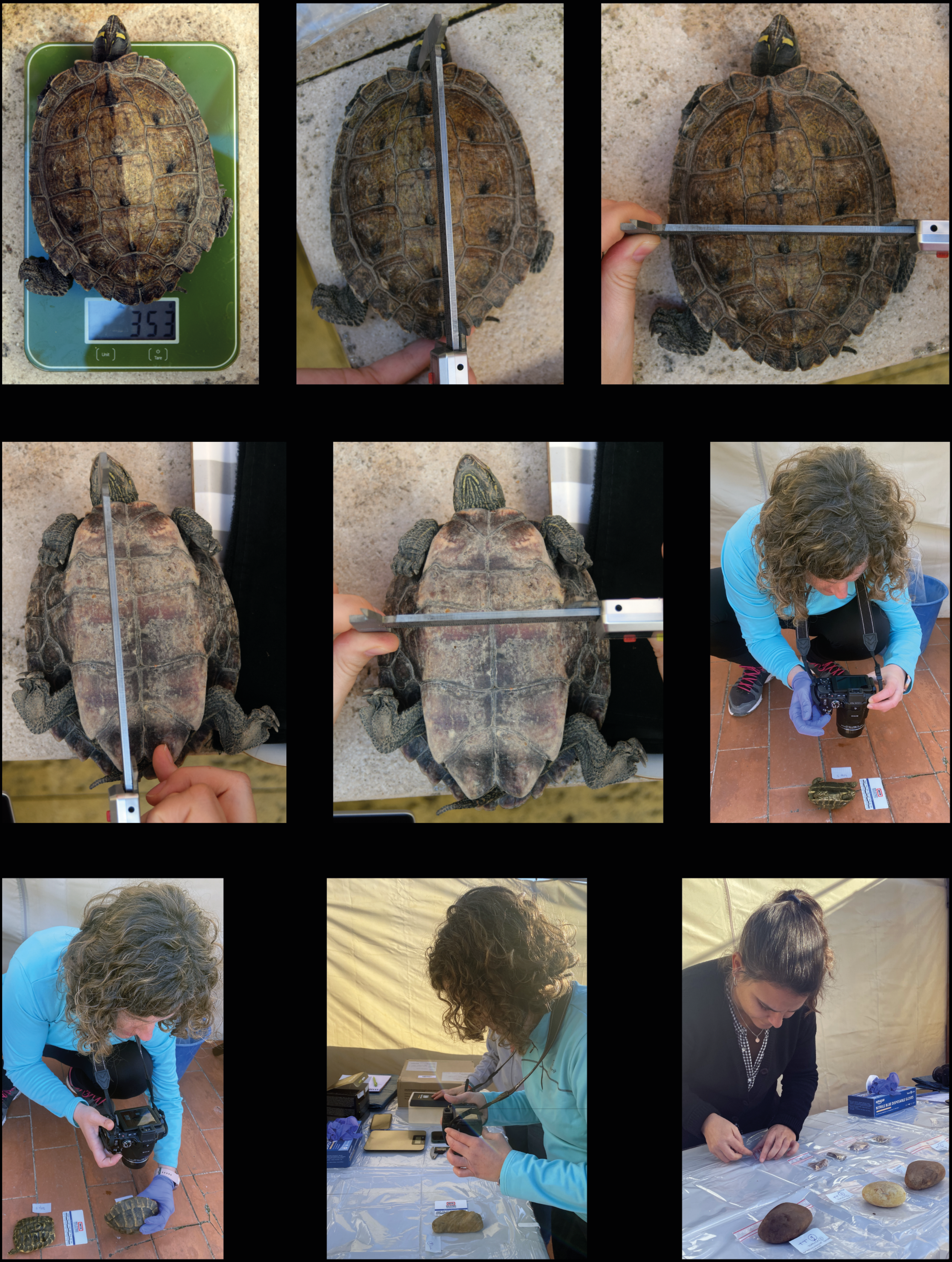

Roasted chelonid processement:

The example of Chelonid 4, *Testudo hermanni*

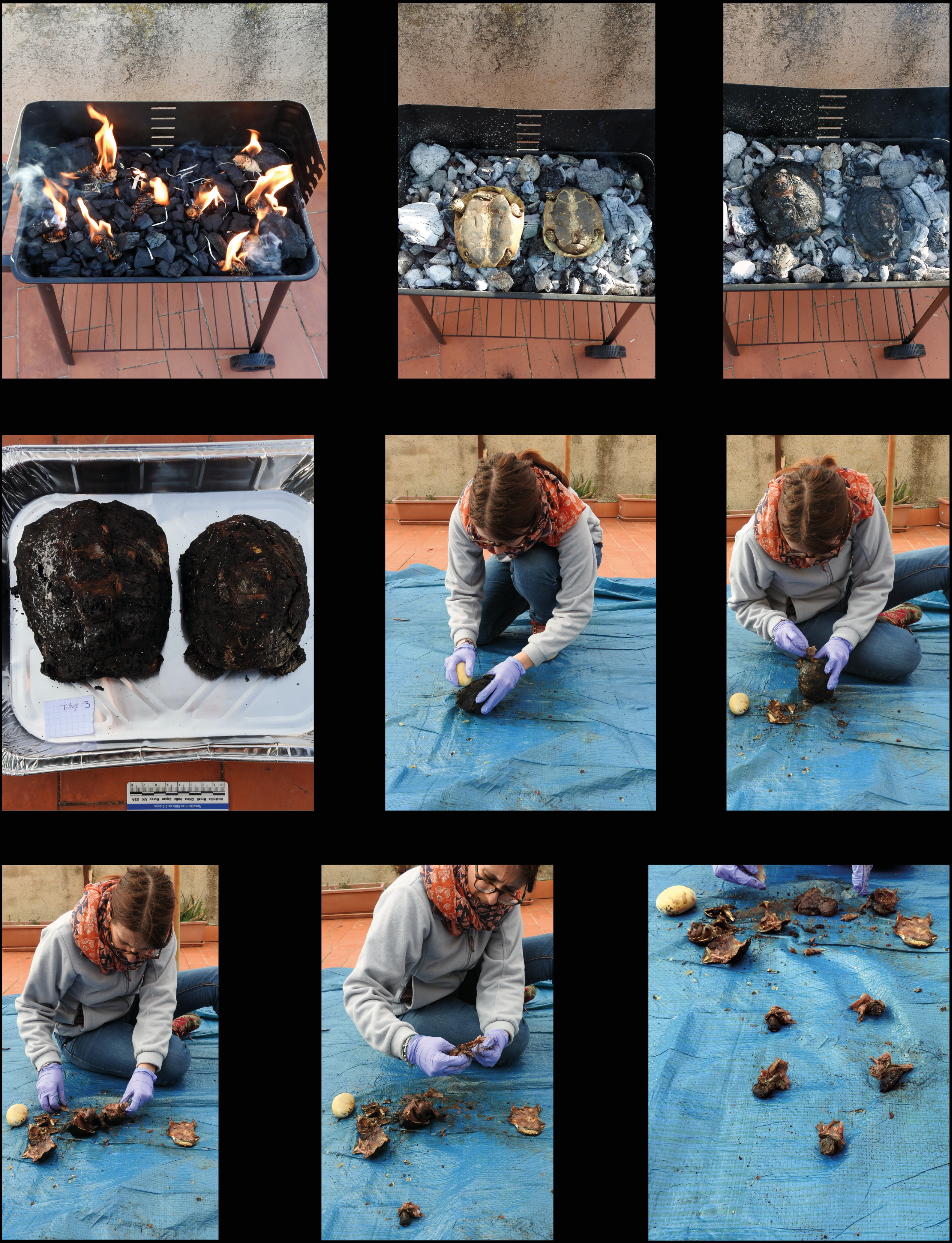

Raw chelonid processement:

The example of Chelonid 6, *Testudo hermanni*

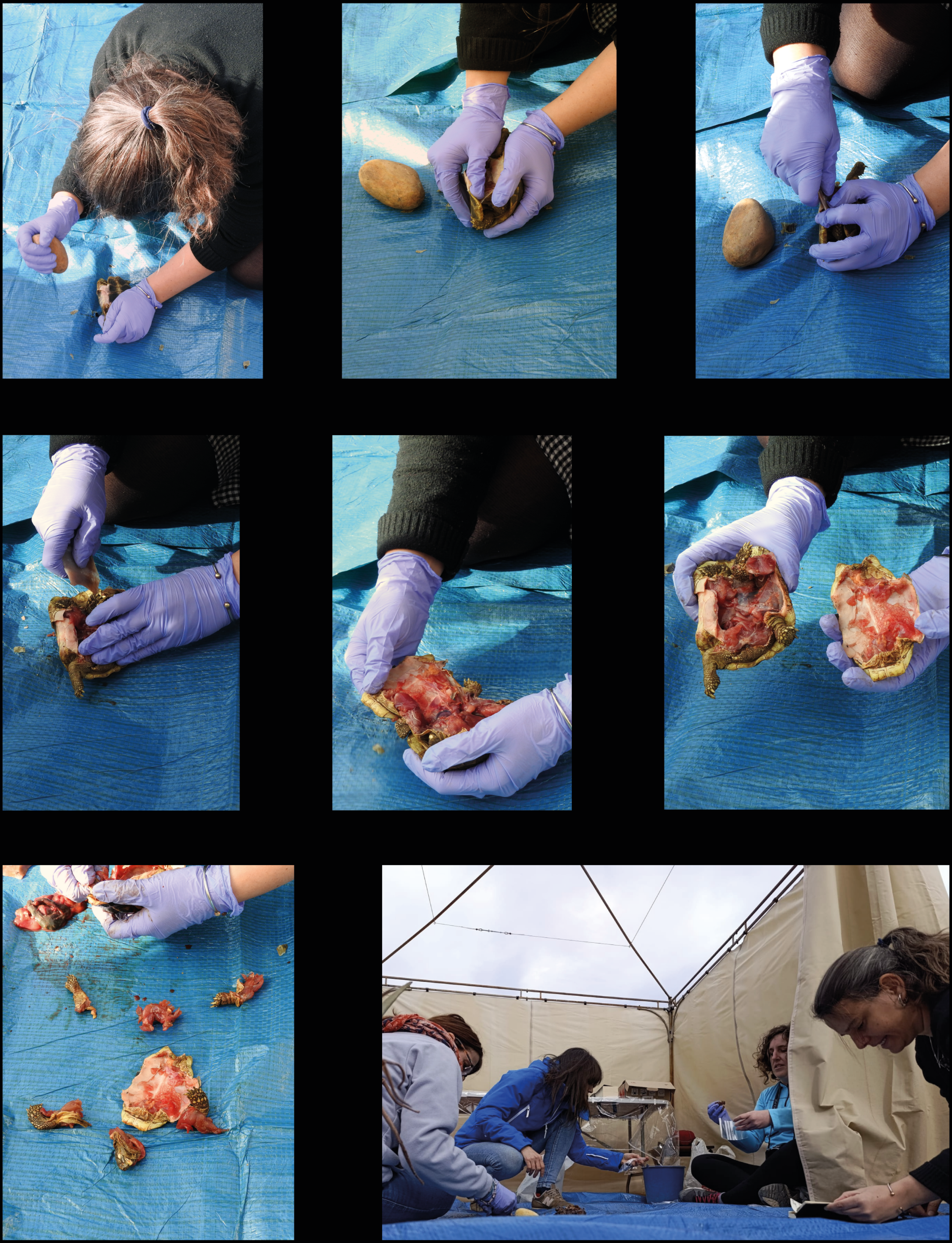

Supplement: Supplementary file 2 — Supplementary Information 2. [file 41598_2025_31738_MOESM2_ESM.pdf]
