# Supplementary material for: Experimental analysis of roasted and raw turtle butchery and implications for early human cognition and behaviour
Source: Sci Rep. 2025 Dec 24;16:1913. doi: 10.1038/s41598-025-31738-z (PMC12804910; doi:10.1038/s41598-025-31738-z)

CHELONID 1
Graptemys ouachitensis

PERCUSSION MARKS
Notches
Impact flakes

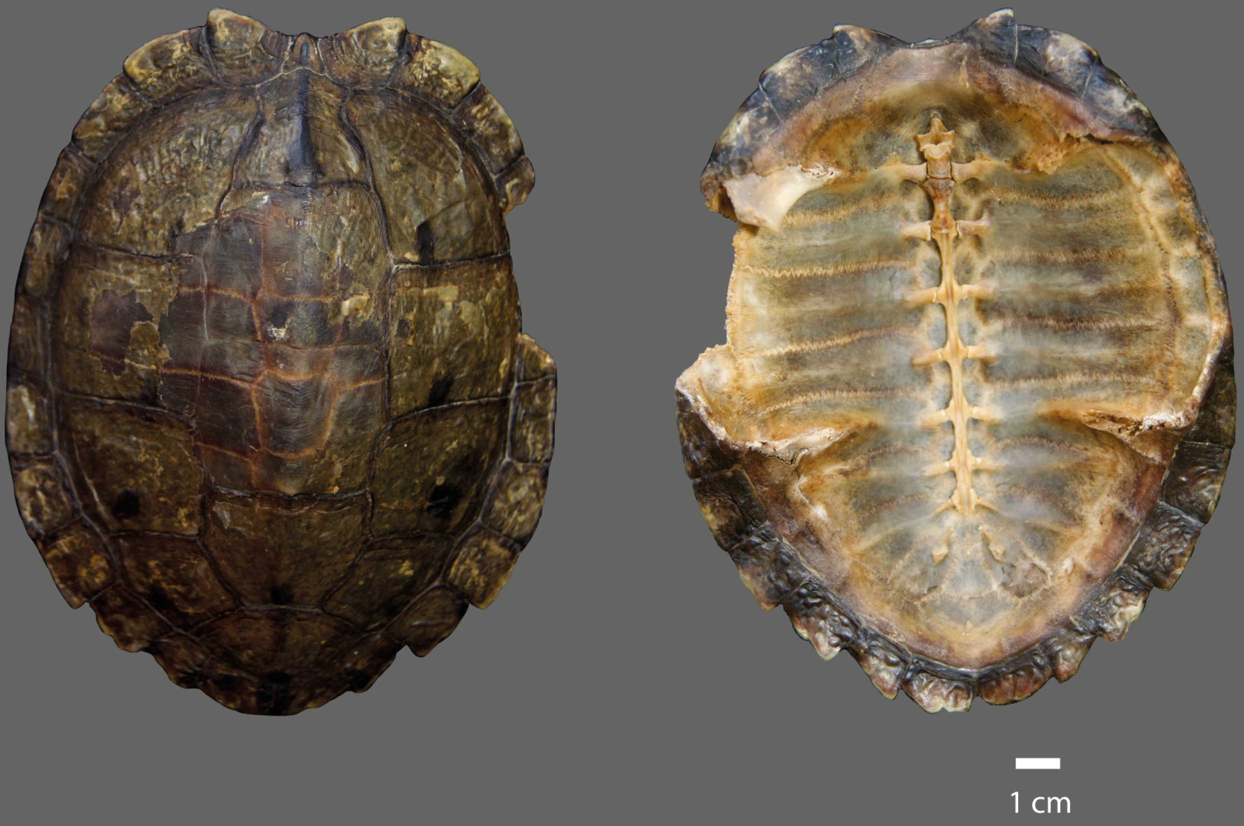

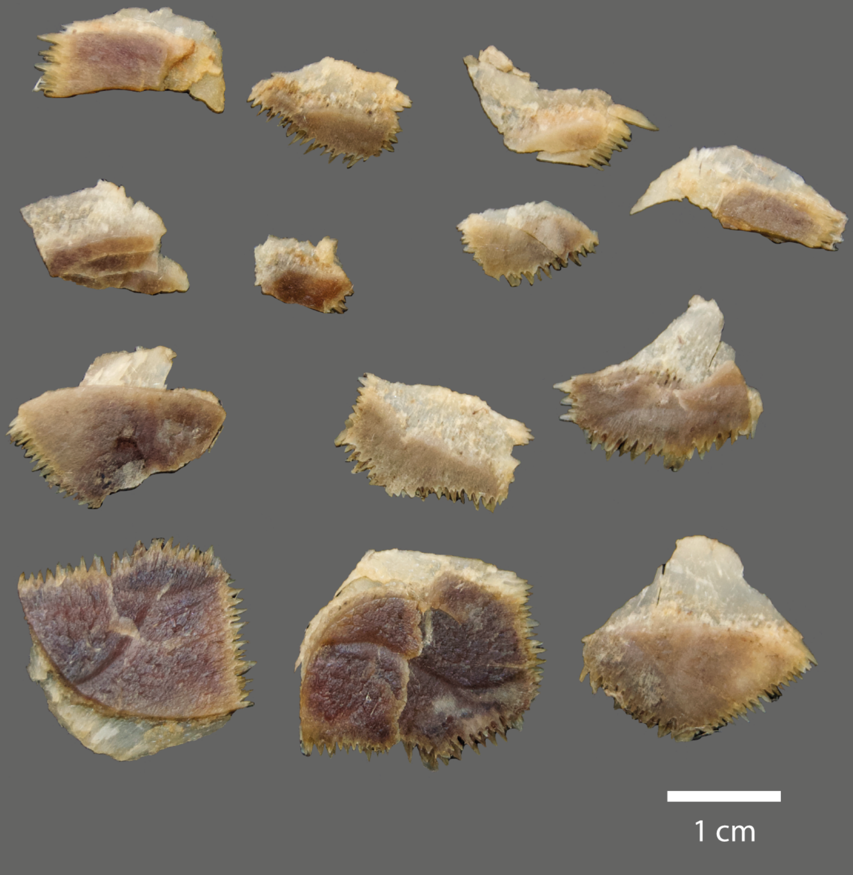

CHELONID 2
Mauremys cf. *reevesii*

BURNING MARKS
Carapace exterior (left)
Plastron exterior (top right)
Plastron interior (bottom right)

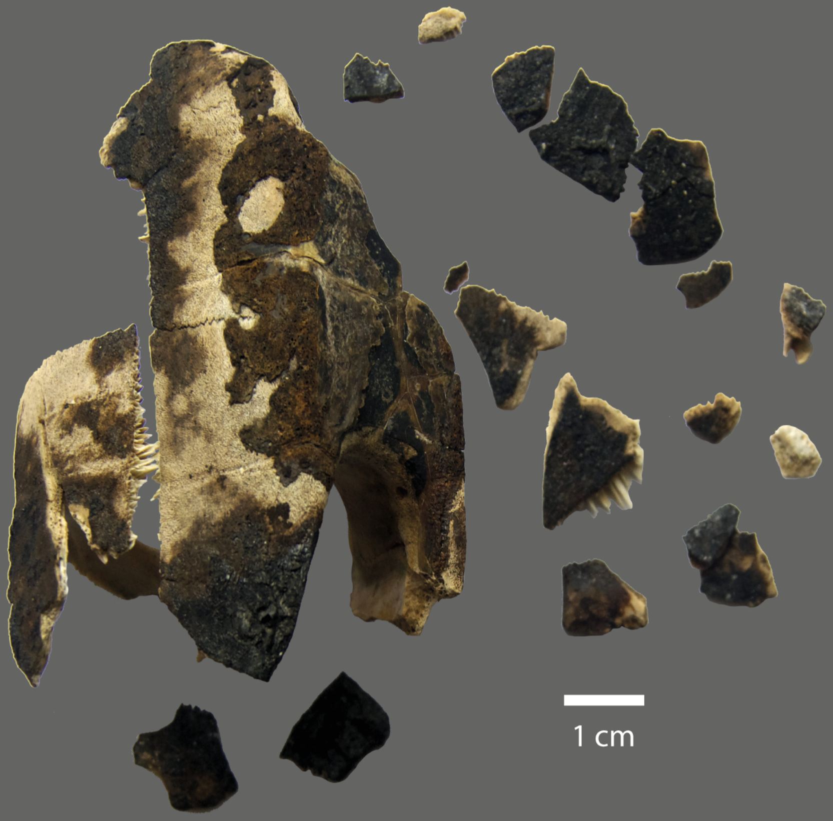

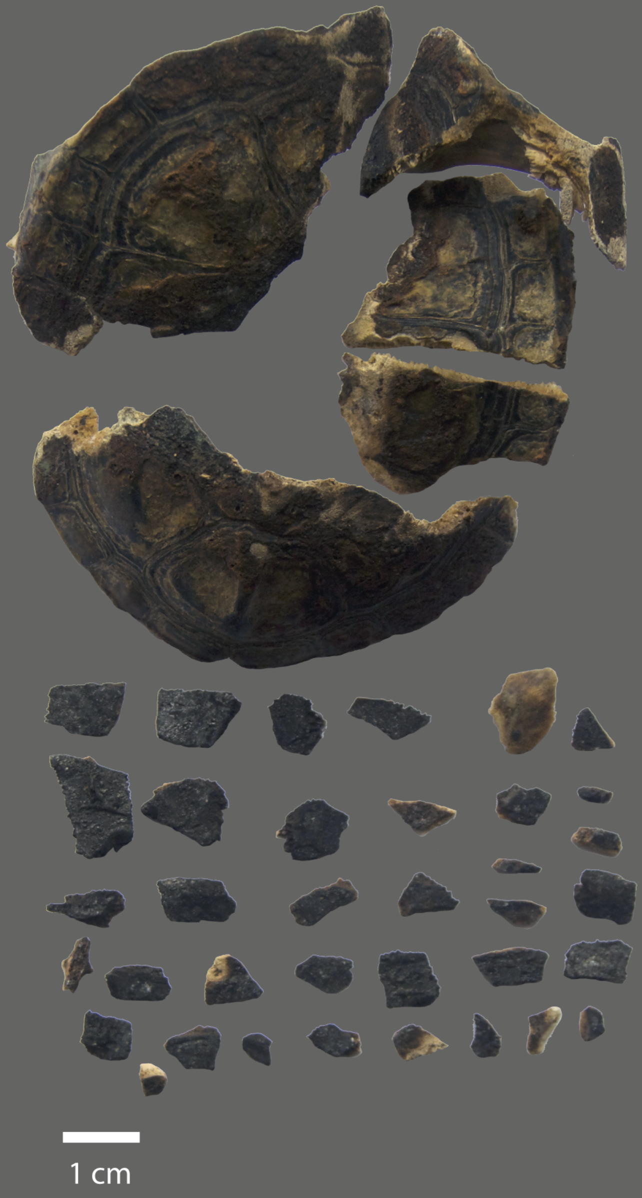

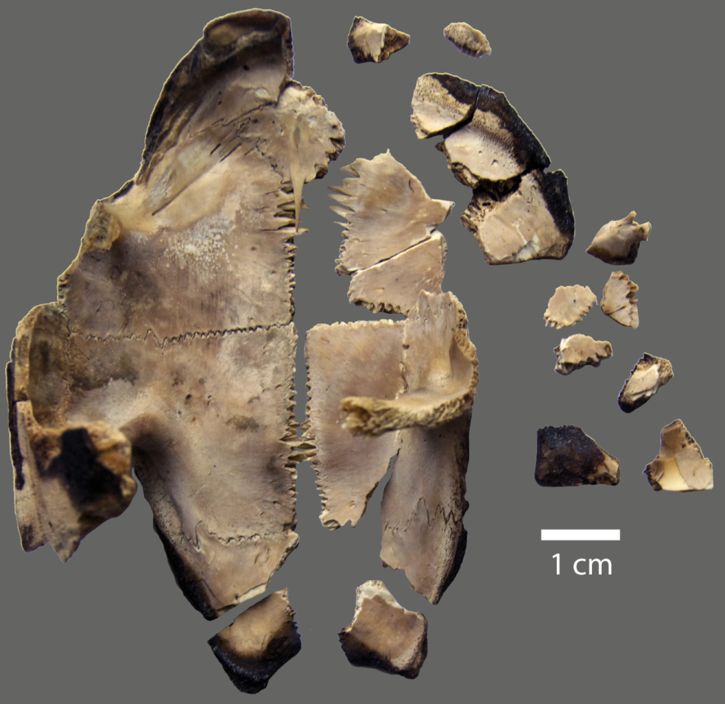

CHELONID 4

Testudo hermanni

BURNING MARKS

Carapace exterior

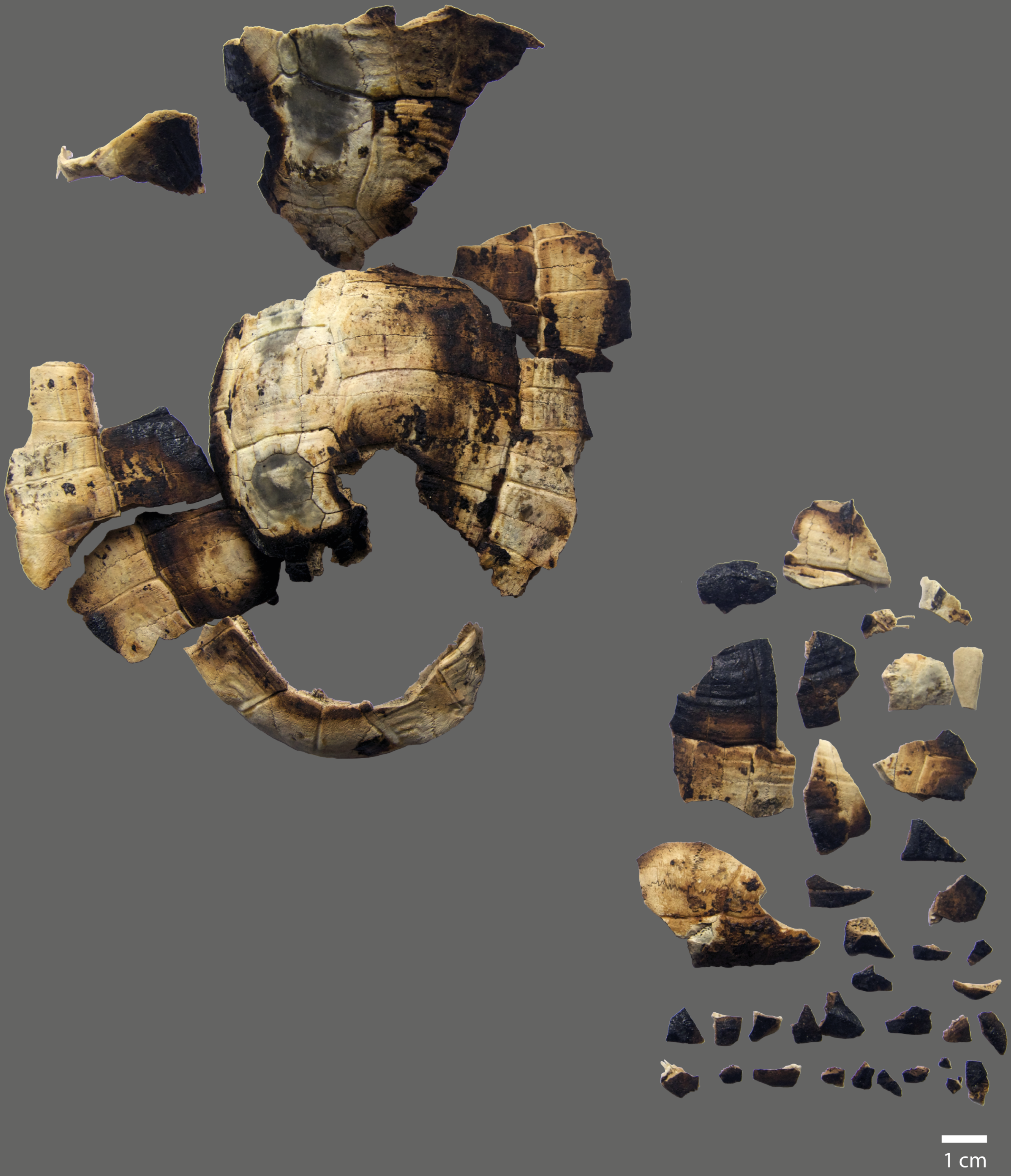

Supplement: Supplementary file 3 — Supplementary Information 3. [file 41598_2025_31738_MOESM3_ESM.pdf]
