# Supplementary figures and images for: Experimental analysis of roasted and raw turtle butchery and implications for early human cognition and behaviour
Source: Sci Rep. 2025 Dec 24;16:1913. doi: 10.1038/s41598-025-31738-z (PMC12804910; doi:10.1038/s41598-025-31738-z)

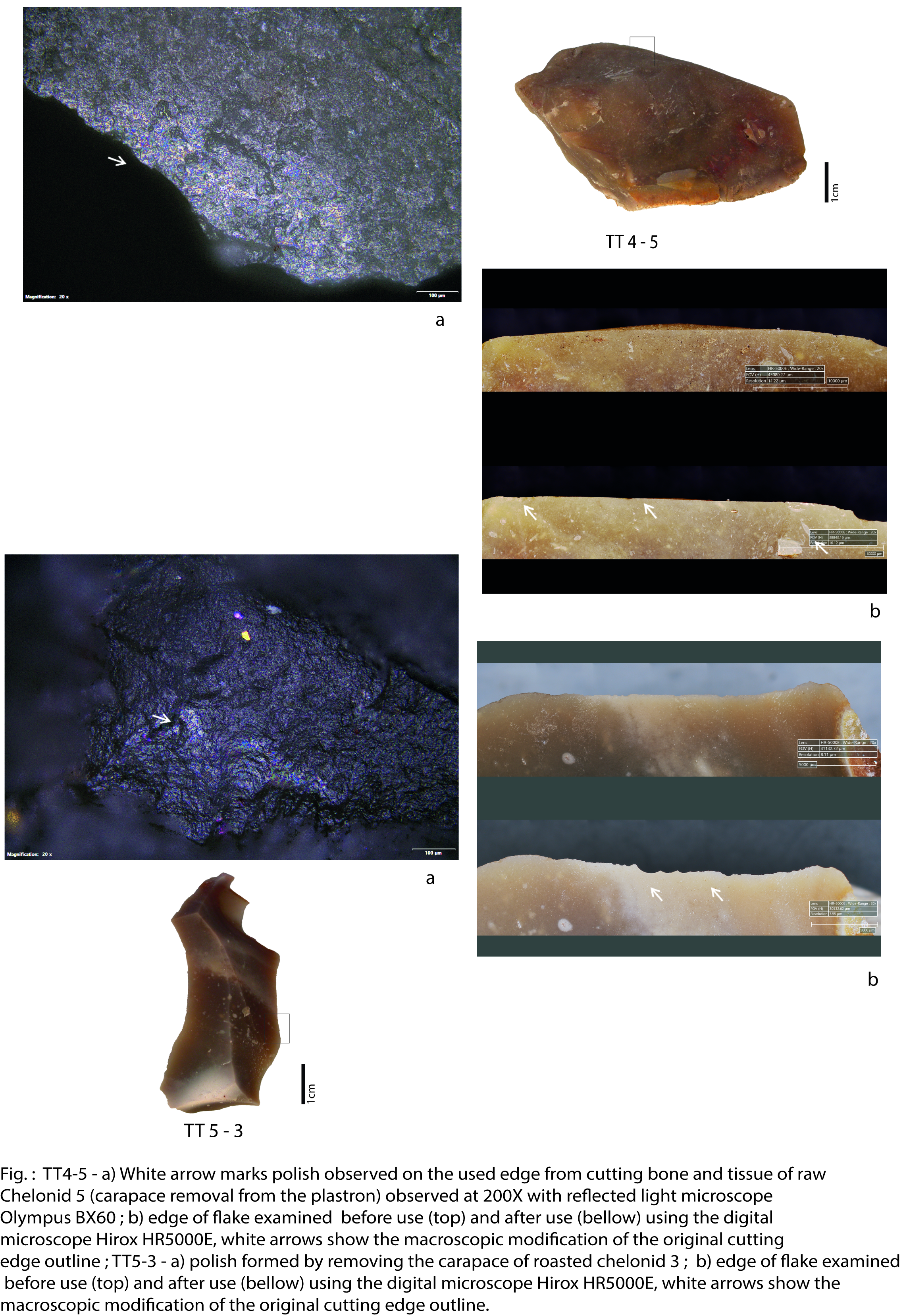

Supplement: Supplementary file 4 — Supplementary Information 4. [file 41598_2025_31738_MOESM4_ESM.tif]
